# The *G2-Like* gene family in *Populus trichocarpa*: identification, evolution and expression profiles

**DOI:** 10.1186/s12863-023-01138-1

**Published:** 2023-07-05

**Authors:** Ruihua Wu, Lin Guo, Yueyang Guo, Lehang Ma, Kehang Xu, Boyu Zhang, Liang Du

**Affiliations:** 1 College of Biological Sciences and Technology, Beijing Forestry University, Beijing, 100083 China; 2 Key Laboratory of Genetics and Breeding in Forest Trees and Ornamental Plants, Ministry of Education, Beijing Forestry University, Beijing, 100083 China

**Keywords:** *Populus trichocarpa*, *GLK* genes, Phylogenetic relationship, Motif analysis, Expression profiles

## Abstract

The *Golden2-like* (GLK) transcription factors are plant-specific transcription factors (TFs) that perform extensive and significant roles in regulating chloroplast development. Here, genome-wide identification, classification, conserved motifs, *cis*-elements, chromosomal locations, evolution and expression patterns of the *PtGLK* genes in the woody model plant *Populus trichocarpa* were analyzed in detail. In total, 55 putative *PtGLKs* (*PtGLK1*-*PtGLK55*) were identified and divided into 11 distinct subfamilies according to the gene structure, motif composition and phylogenetic analysis. Synteny analysis showed that 22 orthologous pairs and highly conservation between regions of *GLK* genes across *P. trichocarpa* and *Arabidopsis* were identified. Furthermore, analysis of the duplication events and divergence times provided insight into the evolutionary patterns of *GLK* genes. The previously published transcriptome data indicated that *PtGLK* genes exhibited distinct expression patterns in various tissues and different stages. Additionally, several *PtGLKs* were significantly upregulated under the responses of cold stress, osmotic stress, and methyl jasmonate (MeJA) and gibberellic acid (GA) treatments, implying that they might take part in abiotic stress and phytohormone responses. Overall, our results provide comprehensive information on the *PtGLK* gene family and elucidate the potential functional characterization of *PtGLK* genes in *P. trichocarpa*.

**Supplementary Information:**

The online version contains supplementary material available at 10.1186/s12863-023-01138-1.

## Introduction

Chloroplasts are responsible for the light energy response of photosynthesis, and contain the green pigment chlorophyll, which is basically relied upon by all plant life [1, 2]. Recent research has shown that chloroplasts originated from primary endosymbiotic events related to these cyanobacteria [3, 4]. Thus, the regulation of photosynthetic organs assembly depends on the synergy of the nucleus and chloroplast. Plastids not only function in photosynthesis but also in the synthesis of amino acids, fatty acids, purines and pyrimidine bases, terpenes and various pigments, and hormones, as well as the key aspects of nitrogen and sulfur assimilation [5–7]. Moreover, proplastids in subepidermal meristem cells (or leaf sheaths in dark cotyledons) convert to mesophyll chloroplasts under light [8]. Conversely, members of the Golden 2-like (GLK) family can regulate the appearance of chloroplasts in the transition and maturity stages, and *GLK* genes are essential in angiosperm chloroplast development [2, 9, 10]

GLK transcription factor was first identified in maize (*Zea mays L.*), and was proven to be a new transcriptional regulator that functions on cellular differentiation in the leaves of maize [11]. The *GLK* genes belong to the GARP superfamily of nuclear transcription factors [12], which are defined by GOLDEN2 in maize, RESPONSE REGULATOR-B (ARR-B) proteins in *Arabidopsis* [13], and the PHOSPHATE STARVATION RESPONSE1 (PSR1) protein in Chlamydomonas [14]. Most GLK proteins contain two domains: a Myb-DNA-binding domain (DBD; containing a helix-loop-helix motif) and a C-terminal box (containing a GCT box) [15, 16].

*GLK* genes are crucial for the formation and development of chloroplasts, and participating in various biotic and abiotic stress defense processes of organisms [17, 18]. In *Arabidopsis*, *AtGLK1* and *AtGLK2* genes were found to be involved in the production of chloroplast redundantly [19, 20]. Overexpression of *AtGLK1* can cause resistance to *Fusarium graminearum* [21, 22] and improve sensitivity on the virulent oomycete pathogen *Hyaloperonospora arabidopsidis* (Hpa) [1]. In addition, *SlGLK2* affects the photosynthesis of developing fruits and contributes to the characteristics of mature fruits in tomato (*Solanum lycopersicum*) [23]. Moreover, owing to the increased expression of chloroplast development and fruit-photosynthesis-related genes, the carbohydrates and carotenoids in ripe fruit were found to be enhanced in the overexpression of *SlGLK2* [24]. *ZmGLK1* is considered as a regulator of the development of chloroplasts in mesophyll cells of C4 tissues, while *GLK* gene pairs plays a redundant role in C3 species and promote the development of chloroplasts in maize [14, 16].

Poplar is an important model plant in the study of woody plants, with the characteristics of rapid growth and easy genetic transformation. The accomplishment of the poplar genome sketch provides potential in gene identification and gene function analysis. The *GLK* genes have been identified and described in maize [25], *Arabidopsis* [17], tomato [26], tobacco [27], and moso bamboo [28]. Nevertheless, there has been no comprehensive study on the *GLK* family genes of *P. trichocarpa*. In this study, 55 putative *PtGLK* genes were identified and classified into 11 groups, taking maize *GLKs,* Arabidopsis *GLKs*, and their conserved domains as references. A comprehensive bioinformatics analysis was carried out to study gene structure, domain composition, chromosome distribution, syntheses analysis, and expression patterns. Promoter *cis*-elements and expression level of genes in response to abiotic stress (cold and osmotic) and phytohormone (MeJA and GA) treatments were also examined. The information derived from this study offers a valuable resource for further study on the characterization and function of the poplar GLK gene family.

## Materials and methods

### Plant material treatment and gene expression analysis

The material used in this study was poplar 84 K (*Populus alba* × *Populus glandulosa*) which is an aspen hybrid poplar from Korea. *Populus trichocarpa* trees were obtained from Beijing Forestry University poplar nursey planting base, and were grown under the settings of 16 h light and were maintained at 25 °C and 85% relative humidity in a greenhouse in Haidian, Beijing, China (39°56′ N,116°25′ E, 43.5 m above sea level). Three-month-old poplar seedlings were treated with osmotic stress, cold stress, and MeJA and GA treatments. For cold stress, the seedlings were positioned in a 4 °C growth chamber and sampled at 0, 1, 3, 6, 12, and 24 h after stress imposition. For osmotic stress, the seedlings were accumulated after being sprayed with 25% polyethylene glycol (PEG) 6000. For phytohormone treatments, a solution of 200 µM jasmonic acid (JA) and 200 mg/L gibberellic acid (GA) were sprinkled onto poplar plants on the basis of the needs and sampled randomly after the phytohormone treatments were applied. Seedlings irrigated at 28 °C in an artificial growth chamber and sprinkled with MS medium solution were used as controls and were sampled at 0 h.

The primers of the 11 *PtGLK* genes were designed by the NCBI Primer-BLAST tool (http://www.ncbi.nlm.nih.gov/tools/primer-blast/) to amplify 200–250 bp PCR products (Table S4). The heatmap of *PtGLK* gene expression was generated using the Amazing Heatmap module in TBtools for the poplar FM (female catkins, prior to seed release), F (female catkins, post-fertilization), M (male catkins), ML (mature leaf), REF (washed fibrous roots < 0.5 cm diameter from field-grown trees), RTC (roots from plants in tissue culture), G43h (seedlings were germinated 43 h post-imbibition), ApB (actively growing shoot apex), AxB (axillary bud), YFB (newly initiated female floral buds), YMB (newly initiated male floral buds), Xylem1(developing phloem), Phloem3 (developing phloem/cambium), and PC (phloem, cortex, epidermis) [29, 30].

### Identification of PtGLKs

Poplar GLK sequences were acquired from the Phytozome12.1 database (https://phytozome.jgi.doe.gov). The previously reported GLK protein sequences of *Arabidopsis* [19] were used for the purpose of identifying the poplar GLK proteins for a BLAST alignment of the poplar protein database. More than 30% similarity and an E-values below 0.001 were set as the parameters to determine the poplar candidate GLK proteins. Then the domains of all poplar GLK proteins were investigated using Pfam (http://pfam.xfam.org/) to determine the putative proteins. The gene IDs, physical positions, sequences of the genes and proteins, and the coding sequences (CDS) were downloaded from the *P. trichocarpa* genome database (https://genome.jgi.doe.gov/portal/Poptr1/Poptr1.home.html). The detailed physical parameters of *PtGLK* genes, including molecular weight (MW) of amino acids, isoelectric point (pI), and length of the CDS, were predicated using ExPASy (http://www.expasy.ch/tools/pi_tool.html) [31].

### Multiple sequence alignment and phylogenetic analysis

The protein sequences of poplar GLK proteins were aligned with the ClustalW tool [32]. The alignment of the PtGLK-domain-containing sequence was displayed by DNAMAN 8 platform (https://www.lynnon.com/dnaman.html). The phylogenetic tree based on the complete PtGLK sequences and the combined phylogenetic tree of GLK protein sequences from *P. trichocarpa*, *Z. mays*, and *Arabidopsis* were constructed with MEGA 7.0.

### Gene structure

The exon/intron structures of *PtGLK* genes were decided by the Gene Structure Display Server (GSDS) platform (http://gsds.cbi.pku.edu.cn/) using the complete genomic sequence and CDS [33]. The conserved motifs presented in PtGLK proteins were analyzed by the online MEME tool (http://meme-suite.org/tools/meme) [34] according to the following rules: optimum width of motifs at 10–50, and maximum number of motifs at 10 residues for PtGLK proteins. Motif annotation was identified using the Pfam tools. The predict protein homology model was analyzed using the Phyre2 website (http://www.sbg.bio.ic.ac.uk/phyre2/html/page.cgi?id=index), and alignment of the PtGLK protein sequences was determined via Hidden Markov Models (HMM) [35].

### Chromosomal location, synteny analysis and duplication events

Gene location information was acquired from the *P. trichocarpa* genome database on the basis of the genome annotation file (gff file), and all *PtGLKs* were mapped onto the poplar chromosomes by MapInspect software (http://www.softsea.com/review/MapInspect.html). The possible gene duplication landscape was identified by the Multiple Collinearity Scan Toolkit (MCScanX) software [36]. Segmental duplication and tandem duplication were determined according to the means covered by Wang et al. (2010) [37]. The syntenic maps were subsequently displayed using the Dual Systeny Plotter software (https://github.com/CJ-Chen/TBtools) [38].

Ka and Ks were computerized by KaKs Calculator 2.0 with Clustalx 2.11, and the Ka/Ks ratios were calculated using DnaSP5 to investigate the gene duplication events [39–41].

### Putative promoter region analysis of PtGLK genes

The 2000 bp upstream sequences of 55 *PtGLK* genes were selected as the putative promoter regions to choose the *cis*-elements. The putative *cis*-regulatory elements were identified by PlantCARE (http://www.dna.affrc.go.jp/PLACE/), and those that responded to abiotic stresses and phytohormone treatments were screened out [42, 43].

## Results

### Identification of PtGLK genes in P. trichocarpa

**Fig. 1:**
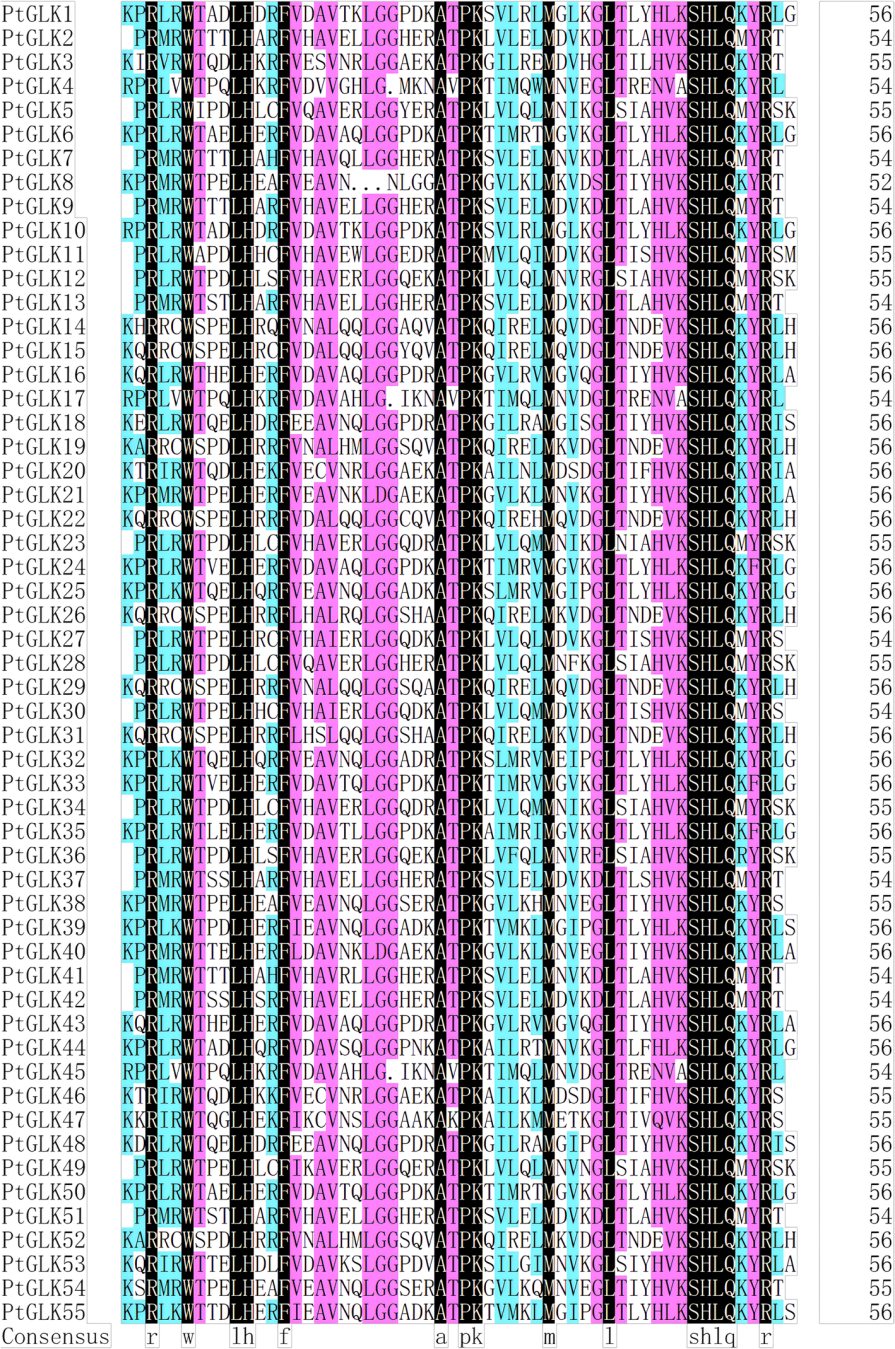
Multiple sequence alignment of the PtGLK conserved domain

To identify the *PtGLK* gene family in *P. trichocarpa*, Arabidopsis AtGLK protein sequences [19] were used as BLASTP sequences in extensive searches and alignment in the poplar genome database. A total of 55 *PtGLK* genes (*PtGLK1*-*PtGLK55*) were identified, all these were used to affirm the existence of the Myb-DNA-binding domain (DBD) through the Pfam database. To further examine the similarity among the PtGLK protein domains, multiple alignments of 55 PtGLK protein domain sequences were conducted (Fig. 1). The result indicated that the *PtGLKs* were conserved across two regions of the Myb-DNA-binding domain with the HLH structure of the first helix containing the initial sequence PELHRR and the second helix containing NI/VASHLQ, which was coincided with the *GLK* members in *Z. mays* [25, 45], *Arabidopsis* [5], tomato [26, 44], tobacco [27], and moso bamboo [28].

Base information about the *PtGLK* genes, such as accession number, gene location, protein length, molecular weight (MW), exon numbers, and physicochemical parameters, is presented in Table 1. The *PtGLK* genes exhibited an inclusive conservation of amino acid sequence lengths and molecular weights. The encoded amino acid sequences ranged from 282 to 486 aa, and the predicted molecular weight (MW) varied from 28.87 to 53.24 kDa. Moreover, the theoretical isoelectric point (pI) ranged from 5.55 to 9.46.

**Table 1:** Detailed information about 55 predicted *PtGLK* genes in *P. trichocarpa*

**Gene name**	**Sequences ID**	**Position**	**MW (Da)**	**PI**	**CDS Length (dp)**	**Size (aa)**	**Exons**
***PtGLK1***	Potri.001G133400	1:10847419-10850001(-)	31627.53	8.81	849	282	6
***PtGLK2***	Potri.001G137600	1:11217022-11222607(-)	42631.70	9.23	1137	378	6
***PtGLK3***	Potri.001G228500	1:24773549-24785927(+)	44752.57	9.46	1194	397	7
***PtGLK4***	Potri.001G243600	1:26128861-26132400(+)	43710.04	5.60	1173	390	1
***PtGLK5***	Potri.001G280000	1:29369697-29372077(+)	40494.88	9.17	1095	364	5
***PtGLK6***	Potri.001G314800	1:32569346-32574020(+)	33183.56	6.19	924	307	6
***PtGLK7***	Potri.002G130200	2:9870763-9874363(+)	37341.82	9.13	1026	341	6
***PtGLK8***	Potri.002G257800	2:24613058-24620174(+)	55859.36	5.67	1527	508	9
***PtGLK9***	Potri.003G096300	3:12224274-12229729(+)	42662.42	8.32	1137	378	6
***PtGLK10***	Potri.003G100100	3:12532374-12535858(+)	31744.78	9.35	852	283	7
***PtGLK11***	Potri.004G010000	4:578584-581272(-)	33504.16	9.29	900	299	6
***PtGLK12***	Potri.004G057900	4:4830585-4835870(-)	44811.55	9.13	1182	393	8
***PtGLK13***	Potri.004G082400	4:6782882-6788780(-)	54009.82	8.91	1461	486	6
***PtGLK14***	Potri.004G144800	4:16752076-16754518(-)	46587.06	6.78	1263	420	5
***PtGLK15***	Potri.005G134600	5:10350601-10352768(+)	40982.06	6.61	1092	363	5
***PtGLK16***	Potri.006G000800	6:65468-69307(-)	36861.30	5.82	1008	335	7
***PtGLK17***	Potri.006G034900	6:2203147-2204103(-)	34337.29	6.29	957	318	1
***PtGLK18***	Potri.006G101000	6:7706396-7709168(+)	29535.26	8.45	774	257	6
***PtGLK19***	Potri.006G155200	6:13942764-13945934(+)	50341.05	6.90	1383	460	4
***PtGLK20***	Potri.006G191000	6:19805840-19809519(-)	46157.24	6.22	1236	411	7
***PtGLK21***	Potri.007G003200	7:217578-223435(+)	52901.02	5.89	1452	483	8
***PtGLK22***	Potri.007G039400	7:3211363-3213546(+)	39293.93	6.39	1056	351	5
***PtGLK23***	Potri.008G071700	8:4407226-4410438(-)	40441.08	6.47	1098	365	5
***PtGLK24***	Potri.008G081800	8:5130397-5132950(-)	39266.66	7.74	1071	356	6
***PtGLK25***	Potri.008G087600	8:5451126-5452933(-)	36023.53	6.28	969	322	6
***PtGLK26***	Potri.008G117500	8:7521481-7524148(+)	41765.06	7.02	1146	381	6
***PtGLK27***	Potri.008G142000	8:9567883-9571945(+)	33287.23	6.41	891	296	6
***PtGLK28***	Potri.009G075100	9:7303554-7305935(-)	36669.94	8.39	984	327	7
***PtGLK29***	Potri.009G106600	9:9281411-9284063(-)	44701.97	7.68	1221	406	5
***PtGLK30***	Potri.010G099600	10:12276444-12280250(-)	33351.41	6.52	894	297	6
***PtGLK31***	Potri.010G128900	10:14543665-14546219(-)	42886.00	7.04	1173	390	5
***PtGLK32***	Potri.010G167901	10:17078789-17080240(+)	36251.75	6.56	984	327	6
***PtGLK33***	Potri.010G174100	10:17485942-17488292(+)	39535.00	8.24	1071	356	6
***PtGLK34***	Potri.010G185700	10:18281593-18283962(+)	40985.52	8.17	1080	366	5
***PtGLK35***	Potri.011G023600	11:1756622-1758677(+)	29795.92	6.90	783	260	6
***PtGLK36***	Potri.011G067150	11:5850100-5859105(-)	45526.25	9.05	1215	404	8
***PtGLK37***	Potri.012G042100	12:3747827-3754998(-)	47609.77	7.74	1311	436	6
***PtGLK38***	Potri.013G048000	13:3422495-3427517(-)	48374.34	5.47	1296	431	7
***PtGLK39***	Potri.013G060200	13:4416769-4421033(-)	46986.79	6.32	1269	422	6
***PtGLK40***	Potri.014G000700	14:86919-101555(-)	46972.34	5.25	1272	423	8
***PtGLK41***	Potri.014G037200	14:2349029-2352687(+)	37514.97	6.76	1029	342	6
***PtGLK42***	Potri.015G031600	15:2434069-2440389(+)	48391.68	8.50	1332	443	6
***PtGLK43***	Potri.016G001100	16:49984-54790(-)	34335.52	5.56	945	314	7
***PtGLK44***	Potri.016G001200	16:54862-58064(-)	33886.38	6.07	945	314	6
***PtGLK45***	Potri.016G032600	16:1838128-1840398(-)	34582.52	6.20	972	323	1
***PtGLK46***	Potri.016G047900	16:3088672-3091778(+)	43015.82	7.20	1143	380	7
***PtGLK47***	Potri.016G048000	16:3093112-3095536(+)	35155.62	8.15	942	313	6
***PtGLK48***	Potri.016G117000	16:12149752-12152009(+)	28871.56	7.14	753	250	6
***PtGLK49***	Potri.016G121800	16:12632080-12634309(-)	42465.18	9.06	1137	378	5
***PtGLK50***	Potri.017G054800	17:4223359-4227988(+)	33428.77	5.79	930	309	6
***PtGLK51***	Potri.017G137600	17:13885991-13891467(+)	53240.15	8.42	1449	482	6
***PtGLK52***	Potri.018G074200	17:8850610-8853341(-)	50668.48	6.66	1392	463	4
***PtGLK53***	Potri.018G151400	18:16178867-16182619(-)	49807.24	6.37	1356	451	8
***PtGLK54***	Potri.019G020900	19:3280854-3285857(-)	48638.99	5.55	1311	436	8
***PtGLK55***	Potri.019G032700	19:4541705-4544412(+)	46728.47	8.46	1263	420	7

### Phylogenetic analysis of the GLK genes and the determination of gene structures

**Fig. 2:**
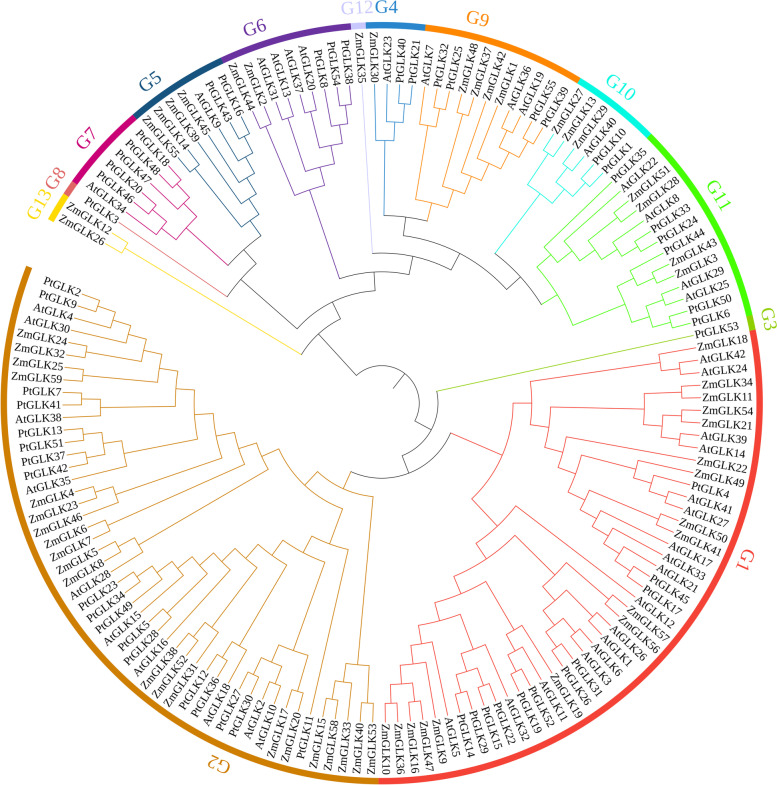
Phylogenetic relationships of GLK proteins of *P. trichocarpa*, *Z. mays*, and *Arabidopsis*. Each specific color represented one group in the branches, and 13 groups were discovered in total

To analyze the evolutionary relationship of the poplar GLK family, a neighbor-joining phylogenetic tree was produced by aligning 55 PtGLK protein sequences with 59 and 42 protein sequences from *Z. mays* [25] and *Arabidopsis*, respectively [5]. The detailed information of *ZmGLK* genes and *AtGLK* genes are listed in Table S1. In the phylogenetic tree, the GLK family members were classed into 13 groups according to the evolutionary relationships and motif analysis of PtGLK proteins, and PtGLKs were assigned into 11 groups (G1-G11), but not G12 and G13. The numbers of PtGLK members in different groups was unbalanced, with groups 1 to 11 containing 11, 18, 1, 2, 2, 3, 2, 5, 5, 2, and 6 proteins, respectively (Fig. 2).

**Fig. 3:**
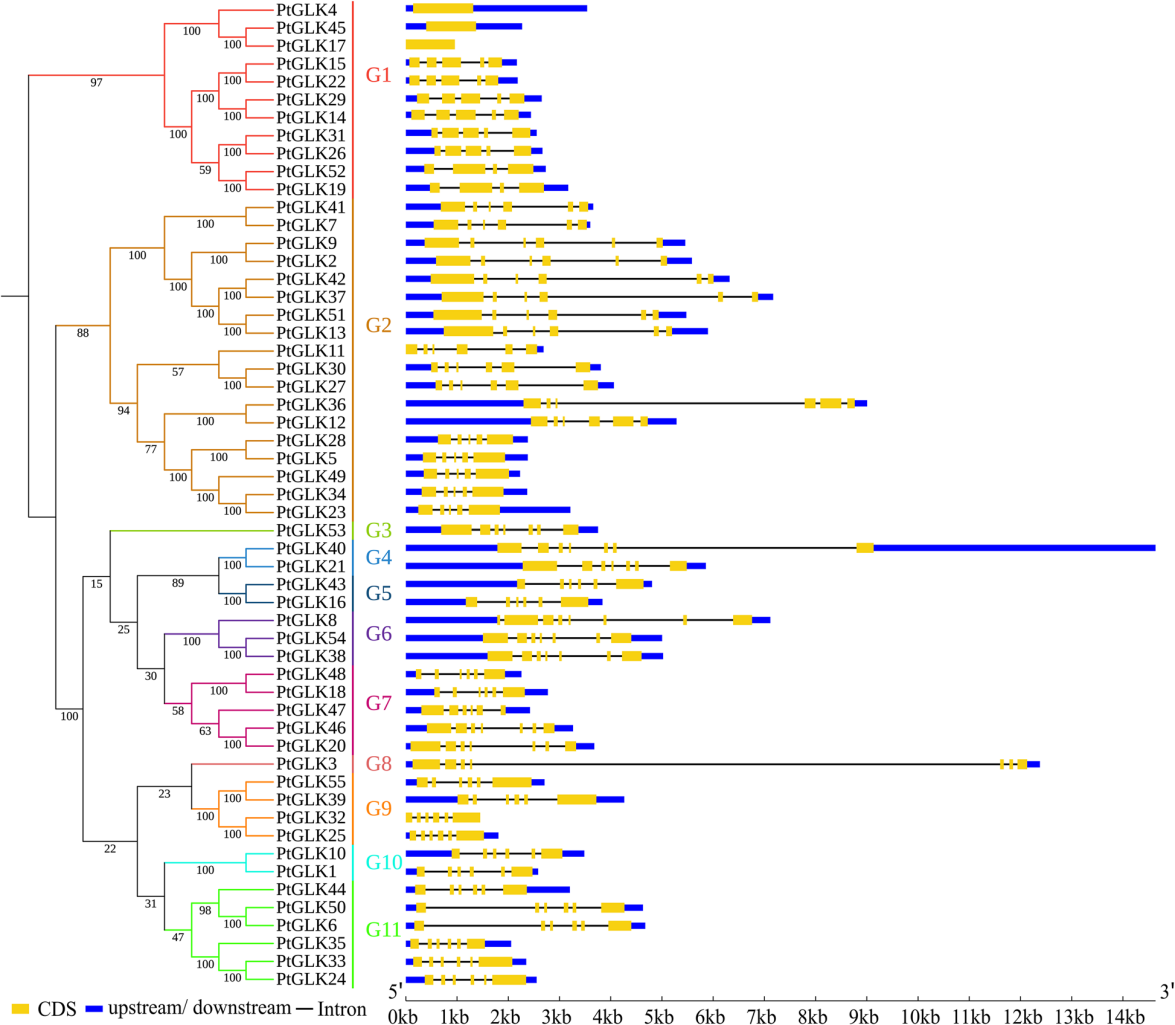
Phylogenetic relationship and exon/intron distribution of *PtGLKs*. The numbers at nodes indicate the bootstrap values per 1000 replicates determined by the neighbor-joining method

A separate phylogenetic tree only with PtGLK proteins was formed to provide additional insight into the structure characteristics of *PtGLK* genes, and all PtGLK proteins were grouped into 11 subfamilies which is consistent with the phylogenetic tree of *P. trichocarpa*, *Z. mays*, and *Arabidopsis*. Exon/intron organization analysis of the *PtGLK* genes, which were defined by the arrangement of *PtGLK* genes, could gain additional insight into the development of poplar GLK family members. The number of exons in the subfamilies ranged from 1 to 9 (Fig. 3). More than half of the *PtGLK* genes (75%) had six or more exons, and only five genes (9%) contained four or fewer exons. The vast majority of the *PtGLK* genes that assembled into the same subfamily exhibited similar or identical exon/intron distributions, including the number of exons and their length. In total, phylogenetic analysis and conservative gene structure provide reliable grouping classification results for *PtGLK* members in the same group. Additionally, the exon/intron structure of each segmentally duplicated pair showed homologous exon/intron distributions.

### Analysis of motif distribution and homology modeling in poplar GLK genes

**Fig. 4:**
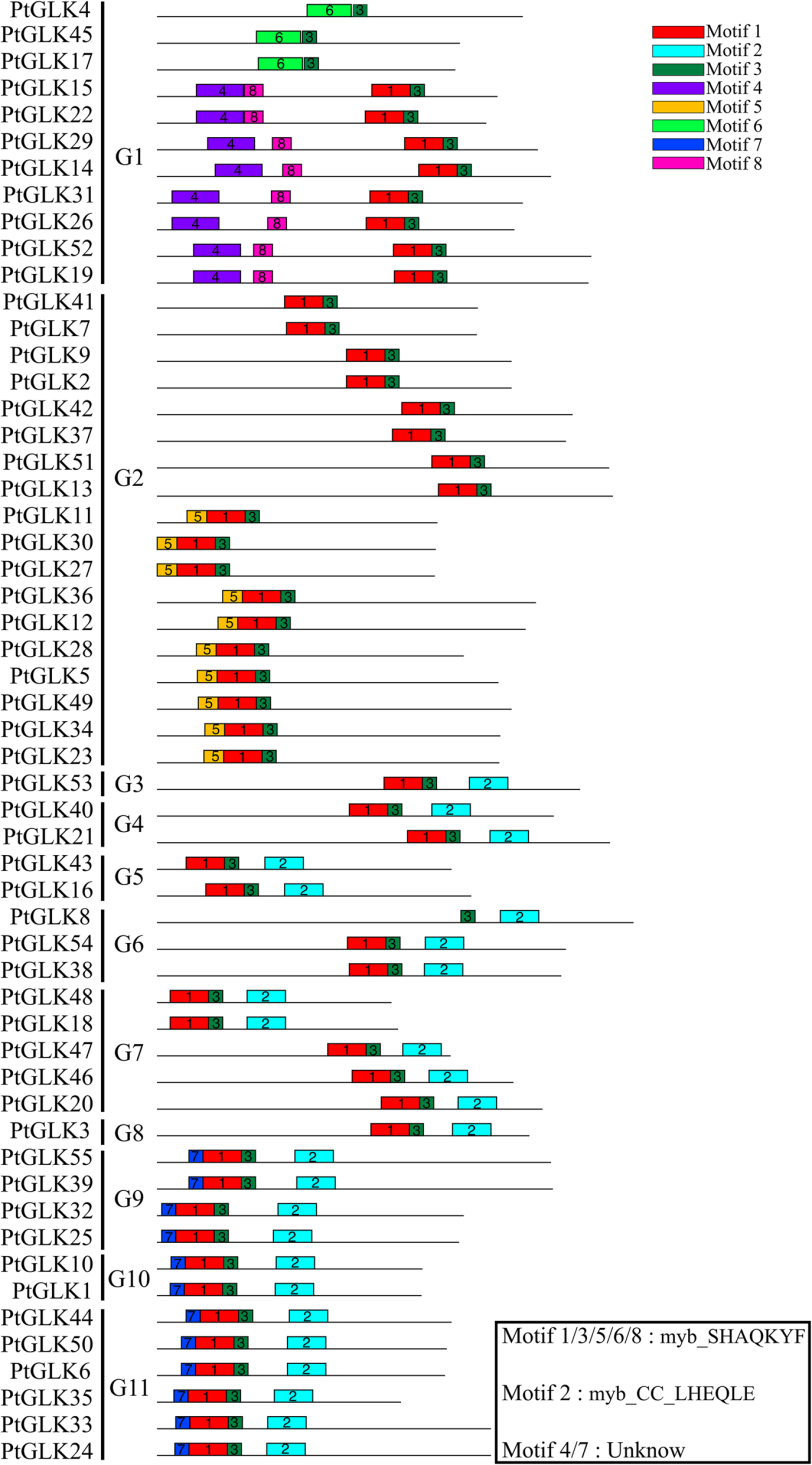
Schematic diagram of 8 conserved motifs (1–8) in PtGLKs, ordered on the basis of online MEME analysis. The 8 motifs are represented in different boxes, and the lengths of the motifs are exhibited proportionally

The conserved motifs of 55 PtGLK proteins within each subfamily were analyzed by MEME software. Eight distinct motifs were identified, and detailed sequence information of each motif is displayed in Table S2. With the Conserved Domain Database, six putative motifs were functional comments, being defined as Myb-SHAQKYF for motifs 1, 3, 5, 6, and 8, and Myb-CC-LHEQLE for motif 2 (Fig. 4). Nevertheless, no functional notes were given to the remaining two putative motifs. Members of the protein family gathered in the same subfamily displayed similar or identical motif components and spatial distributions, which revealed the functional similarities of these proteins. For example, all the PtGLK proteins contained a Myb DNA-binding domain (motif 3), which has an HLH structure. Besides the conserved GLK Myb-DNA-binding domain, the members within different subfamilies had specificity motifs that probably represent their variety functions in plant development and in response to abiotic stress (Figure S1). For instance, motif 2 (Myb-CC-LHEQLE) only appeared in subfamilies 3, 4, 5, 6, 7, 8, 9, 10, and 11.

**Fig. 5:**
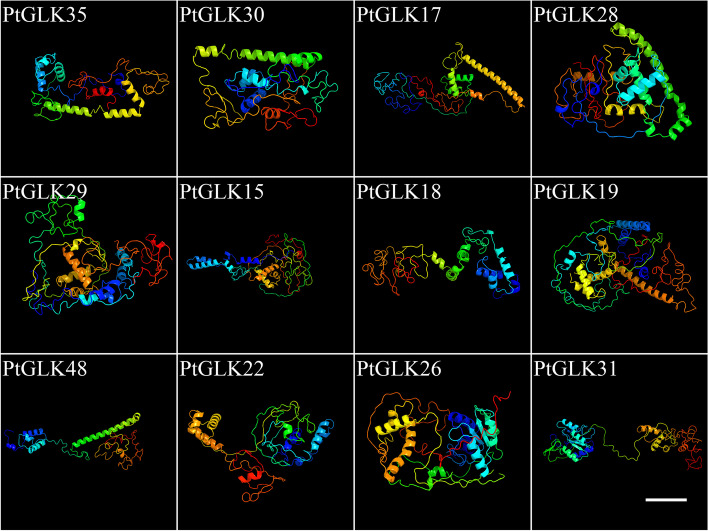
Predicted structures of PtGLK proteins. Bars: 20 nm

To further investigate the potential structures of the PtGLK proteins, we made use of Phyre2 to predict the homology modeling and aligned the protein sequences [45]. The result in Fig. 5 showed that each PtGLK protein could be modeled with confidence, and 12 PtGLKs (PtGLK17, PtGLK18, PtGLK19, PtGLK22, PtGLK26, PtGLK28, PtGLK29, PtGLK30, PtGLK31, PtGLK35, and PtGLK48) among them had 100% of their predicted lengths modeled with > 40% confidence.

### Chromosomal locations and synteny analysis of PtGLK genes in P. trichocarpa

**Fig. 6:**
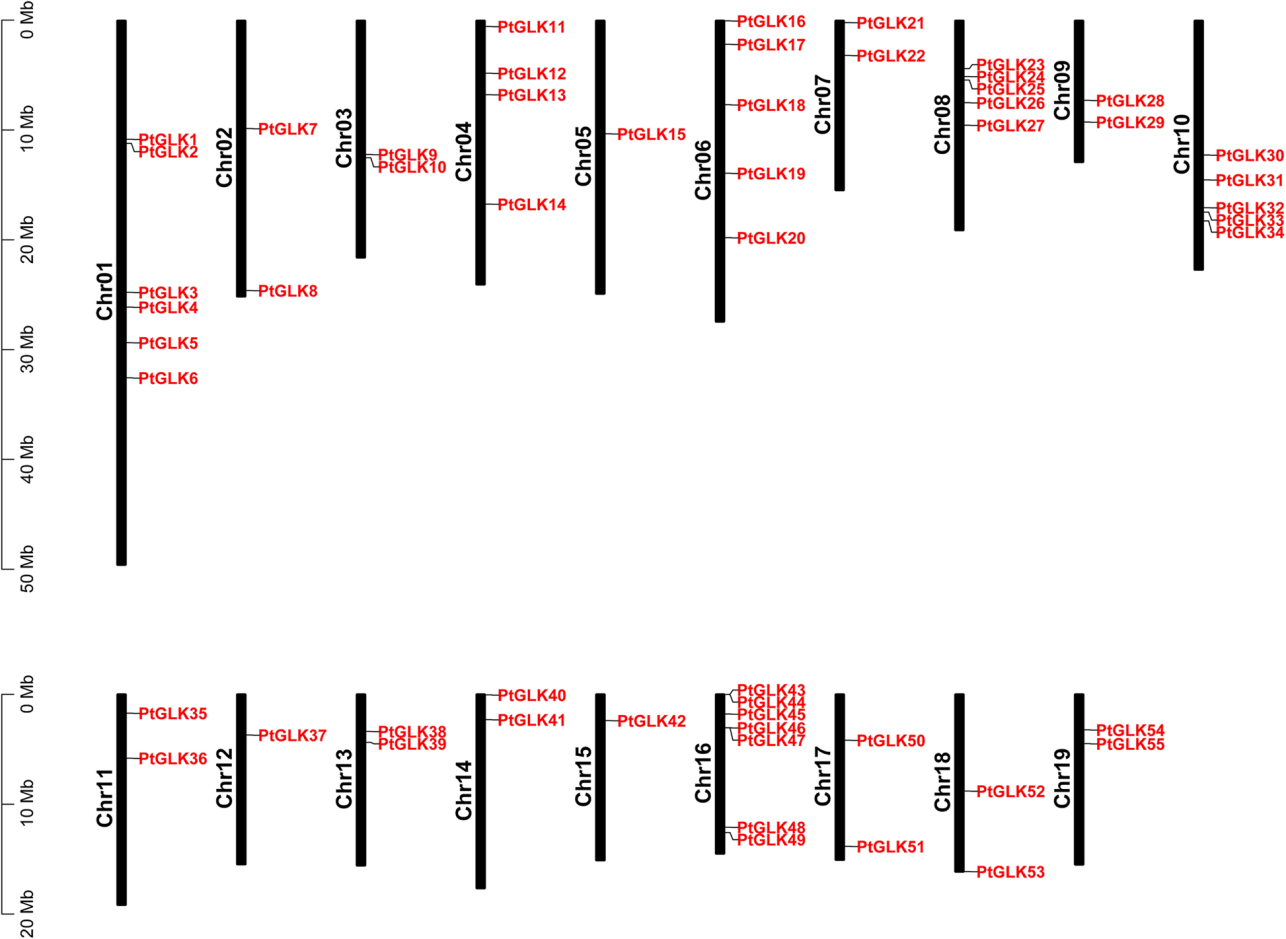
Chromosomal locations of *PtGLK* genes in *P. trichocarpa*

**Fig. 7:**
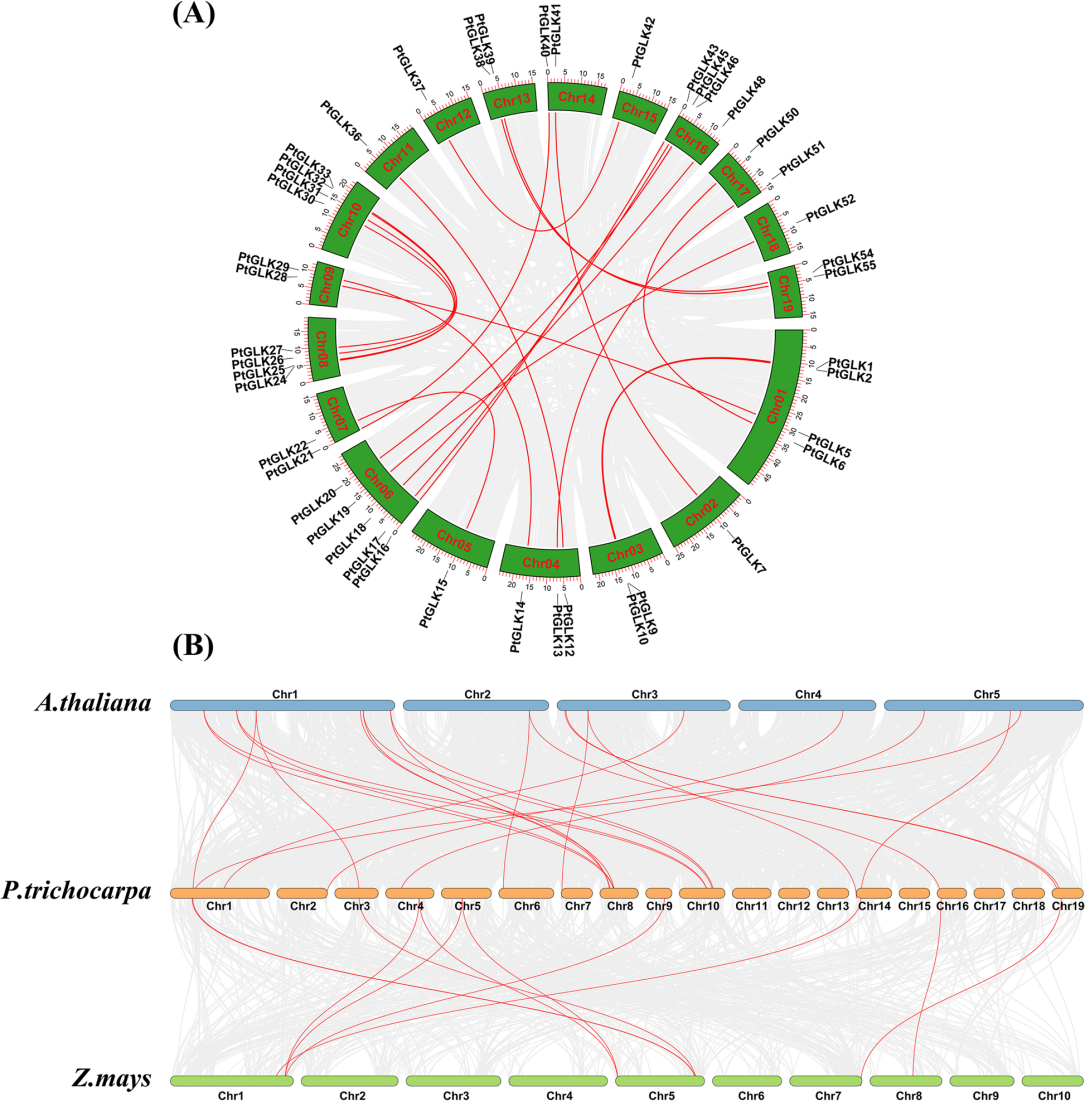
Synteny analysis of GLK proteins. **A** Synteny of *PtGLK* genes in *P. trichocarpa*. **B** Synteny of *GLK* genes between *P. trichocarpa* and two plant species (*Arabidopsis* and *Z. mays*)

A total of 55 *PtGLKs* were acquired and were distributed to the 19 poplar chromosomes (Chr1-Chr19) (Fig. 6). The number of *PtGLKs* per chromosome ranged from one to seven. For example, chromosome 16 contained seven *PtGLK* genes, with the largest number, followed by chromosome 1, with six, and chromosomes 6, 8, and 10 with five. Conversely, chromosomes 12 and 15 possessed only one *PtGLK* gene each. In addition, the potential duplication events were analyzed by the MCScanX program to search the mechanism for the *PtGLK* gene family. A total of 22 duplicated pairs of *PtGLK* genes were defined as segmental duplication gene pairs, but not tandem duplication gene pairs, in a syntenic map (Fig. 7A, and Table 2). Moreover, the analysis showed that there was an unevenly distribution mode among the 22 segmental duplicated pairs on the 19 chromosomes. These results suggested that segmental duplication events probably play a primary role in the amplification of the poplar GLK gene family.

**Table 2:** Ka/Ks of paralogous *PtGLK* gene pairs (Pt–Pt) in *P. trichocarpa*

Paralogous	Ka	Ks	Ka/Ks	Selection pressure	Duplicate type
*PtGLK2/9*	0.07	0.22	0.29	Purifying selection	Segmental duplication
*PtGLK1/10*	0.06	0.21	0.31	Purifying selection	Segmental duplication
*PtGLK5/28*	0.11	0.23	0.50	Purifying selection	Segmental duplication
*PtGLK6/50*	0.03	0.17	0.17	Purifying selection	Segmental duplication
*PtGLK7/41*	0.10	0.16	0.65	Purifying selection	Segmental duplication
*PtGLK12/36*	0.12	0.24	0.49	Purifying selection	Segmental duplication
*PtGLK13/51*	0.05	0.24	0.22	Purifying selection	Segmental duplication
*PtGLK14/29*	0.07	0.23	0.29	Purifying selection	Segmental duplication
*PtGLK15/22*	0.08	0.26	0.31	Purifying selection	Segmental duplication
*PtGLK16/43*	0.04	0.16	0.28	Purifying selection	Segmental duplication
*PtGLK17/45*	0.03	0.24	0.14	Purifying selection	Segmental duplication
*PtGLK18/48*	0.06	0.33	0.18	Purifying selection	Segmental duplication
*PtGLK19/52*	0.06	0.30	0.21	Purifying selection	Segmental duplication
*PtGLK20/46*	0.08	0.30	0.27	Purifying selection	Segmental duplication
*PtGLK21/40*	0.07	0.31	0.23	Purifying selection	Segmental duplication
*PtGLK24/33*	0.05	0.18	0.28	Purifying selection	Segmental duplication
*PtGLK25/32*	0.06	0.18	0.36	Purifying selection	Segmental duplication
*PtGLK26/31*	0.07	0.29	0.25	Purifying selection	Segmental duplication
*PtGLK27/30*	0.09	0.27	0.34	Purifying selection	Segmental duplication
*PtGLK37/42*	0.08	0.24	0.33	Purifying selection	Segmental duplication
*PtGLK38/54*	0.10	0.20	0.50	Purifying selection	Segmental duplication
*PtGLK39/55*	0.07	0.28	0.24	Purifying selection	Segmental duplication

To further determine the evolutionary orthologous relationships of *PtGLKs*, two comparative syntenic maps of *P. trichocarpa* related to *Arabidopsis* and *Z. mays* were also drawn (Fig. 7B). As shown in Table 3, 22 and 11 orthologs of *P. trichocarpa* between *Arabidopsis* (Pt-At) and *Z. mays* (Pt-Zm) were identified, respectively. Moreover, highly conserved microsynteny was found among the regions of *PtGLK* genes between *P. trichocarpa* and *Arabidopsis*, especially in Pt8 and At1 and in Pt10 and At1, with seven and four synteny genes, respectively.

**Table 3:** Ka/Ks of orthologous *GLK* gene pairs (Pt-At) in *P. trichocarpa* and *Arabidopsis*

Paralogous	Ka	Ks	Ka/Ks	Selection pressure
*PtGLK26/AtGLK1*	0.32	1.68	0.19	Purifying selection
*PtGLK31/AtGLK1*	0.33	1.66	0.20	Purifying selection
*PtGLK26/AtGLK3*	0.30	1.95	0.15	Purifying selection
*PtGLK31/AtGLK3*	0.30	2.52	0.12	Purifying selection
*PtGLK2/AtGLK4*	0.33	1.80	0.19	Purifying selection
*PtGLK9/AtGLK4*	0.30	2.46	0.12	Purifying selection
*PtGLK26/AtGLK6*	0.28	3.07	0.09	Purifying selection
*PtGLK25/AtGLK7*	0.31	1.93	0.16	Purifying selection
*PtGLK32/AtGLK7*	0.29	1.73	0.17	Purifying selection
*PtGLK24/AtGLK8*	0.24	1.66	0.15	Purifying selection
*PtGLK33/AtGLK8*	0.22	2.04	0.11	Purifying selection
*PtGLK17/AtGLK17*	0.27	4.41	0.06	Purifying selection
*PtGLK45/AtGLK17*	0.27	3.27	0.08	Purifying selection
*PtGLK55/AtGLK19*	0.25	2.25	0.11	Purifying selection
*PtGLK54/AtGLK20*	0.44	1.82	0.24	Purifying selection
*PtGLK21/AtGLK23*	0.33	2.10	0.16	Purifying selection
*PtGLK40/AtGLK23*	0.35	1.94	0.18	Purifying selection
*PtGLK4/AtGLK27*	0.36	2.29	0.16	Purifying selection
*PtGLK8/AtGLK31*	0.35	1.71	0.20	Purifying selection
*PtGLK13/AtGLK35*	0.33	2.00	0.17	Purifying selection
*PtGLK41/AtGLK38*	0.32	1.84	0.18	Purifying selection
*PtGLK1/AtGLK40*	0.38	3.29	0.12	Purifying selection

### Evolutionary and divergence patterns of the GLK gene family

**Fig. 8:**
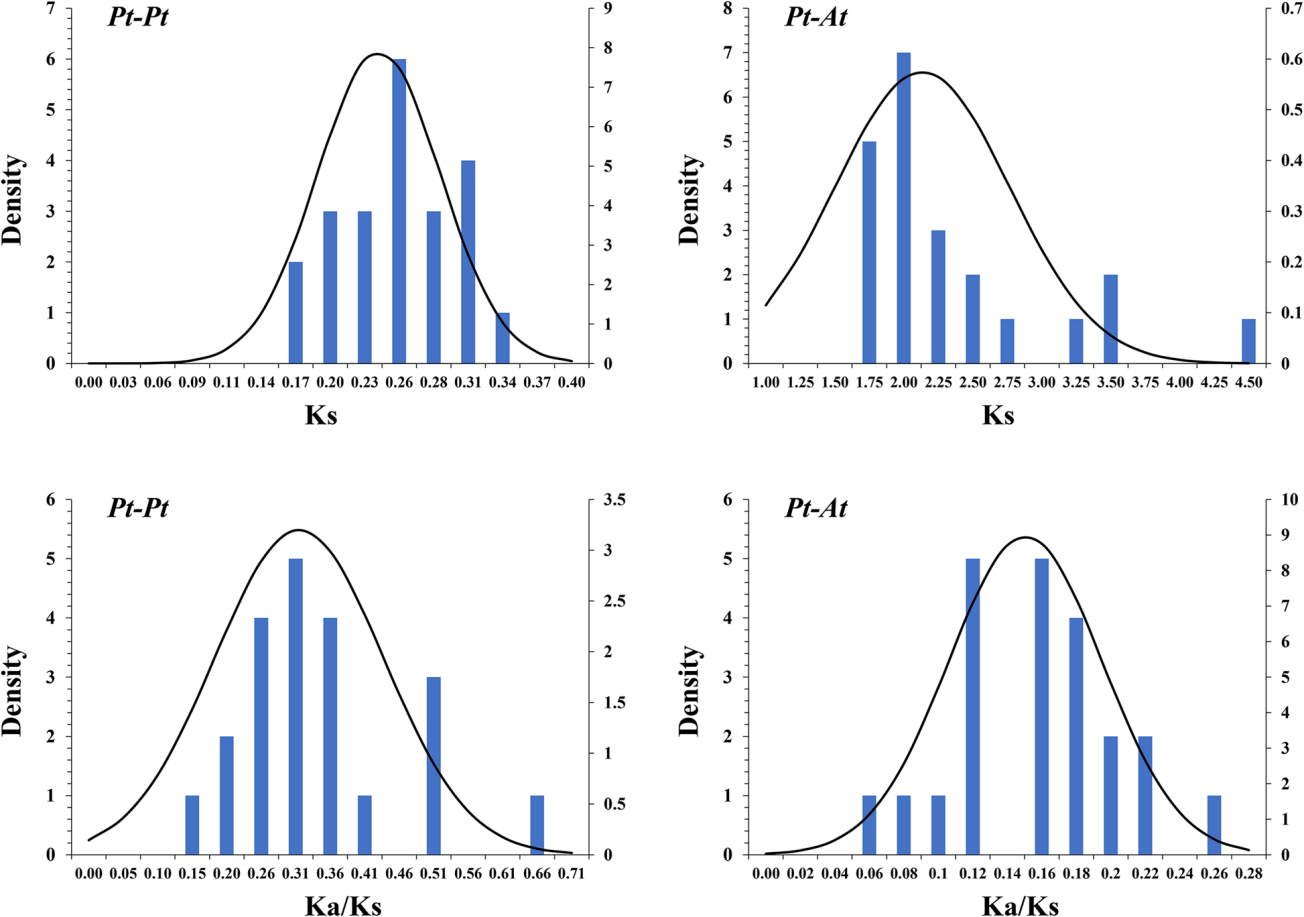
Ks and Ka/Ks value distribution of *PtGLK* genes in paralogous gene pairs (Pt–Pt) of the poplar genome and orthologous gene pairs between *P. trichocarpa* and *Arabidopsis*

For each *PtGLK* gene pair, the Ka/Ks ratios were calculated to evaluate divergence times and selective pressure for the duplicated *PtGLK* genes. To further search the evolutionary events and divergence profiles of *GLK* genes between *P. trichocarpa* and *Arabidopsis*, statistical analysis of the Ka/Ks ratios and the Ks values were conducted. The average frequency distribution of the calculated Ks values of paralogous pairs (Pt–Pt) was approximately 0.24, suggesting that *PtGLK* genes went through a large-scale duplication event was approximately 17 million years ago (MYA) (Fig. 8 and Table 2). Compared with a prior study indicating the timing of a whole-genome duplication in *P. trichocarpa* at 7–12 MYA [46], this result indicated that the large-scale duplication of *PtGLK* genes occurred earlier [41]. Additionally, the frequency distributions of Ks values for the orthologous pairs from the *P. trichocarpa* and *Arabidopsis* genomes averaged ~ 2.25 (Fig. 8, Table 3), suggesting that the divergence time of the *GLK* genes was 118 MYA. With reference to a previous study, it can be inferred that the divergence times between *P. trichocarpa* and *Arabidopsis* were 102–113, this result indicated that the *PtGLK* genes went through gene evolution before the separation of *Z. mays*. The Ka/Ks ratios peak in the poplar genome (Pt–Pt) and between the *P. trichocarpa* and *Arabidopsis* genomes (Pt-At) were distributed between 0.14–0.50 (Table 2) and 0.06–0.20 (Table 3), respectively, which suggests that *PtGLK* genes have probably experienced highly positive purifying selection between *P. trichocarpa* and *Arabidopsis* genomes, as well as being paralogous in the poplar genome.

### Expression profiles of PtGLK genes in various tissues and stages of P. trichocarpa

**Fig. 9:**
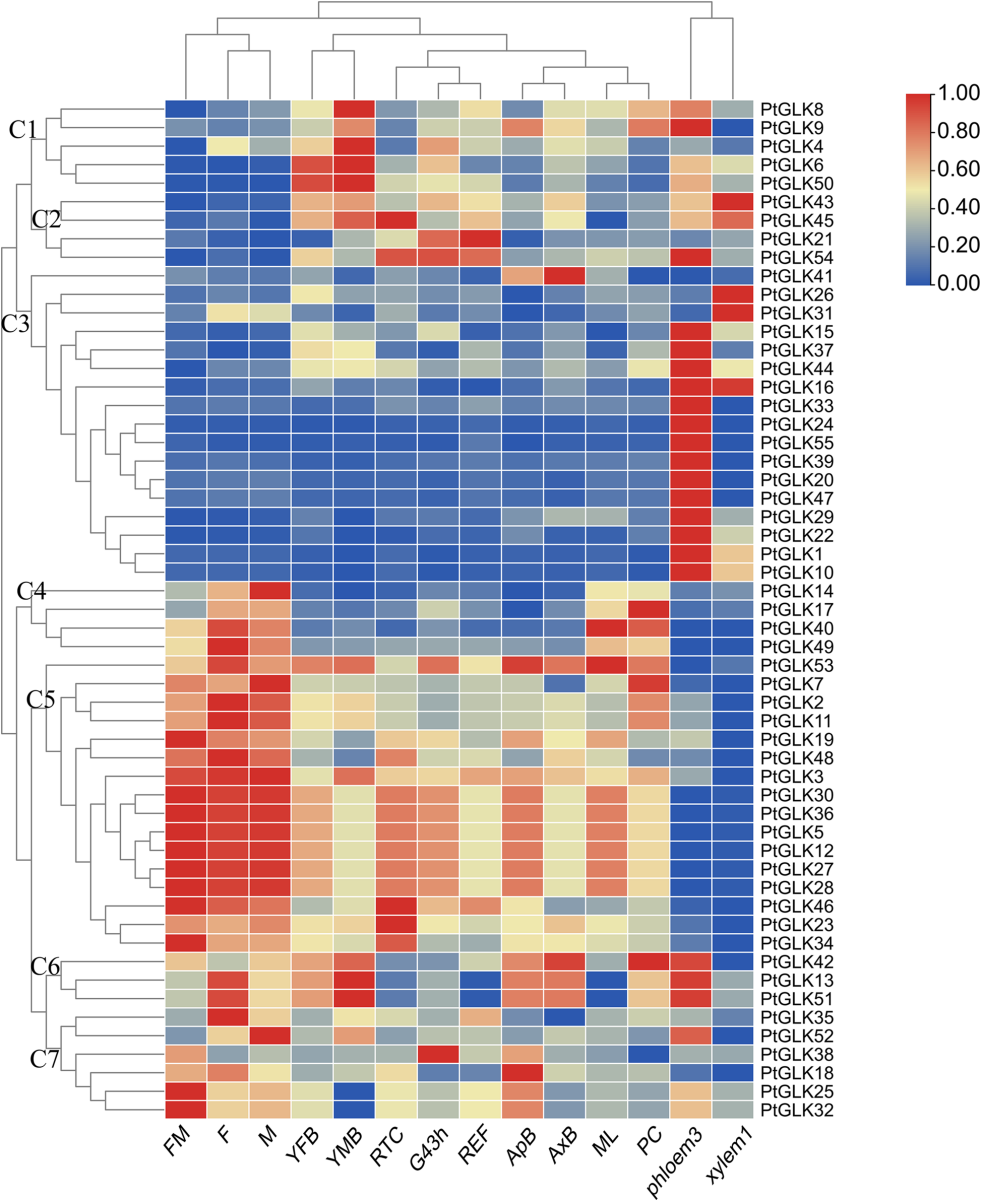
Expression profiles of *PtGLK* genes in different vegetative tissues and stages of reproductive development

To characterize the dynamics of *PtGLK* gene expression, we studied gene expression patterns in several vegetative tissues and stages of poplar reproductive development using high-throughput RNA sequencing (RNA-seq) data from a public database produced in an earlier research [47]. The *GLK* expression patterns were analyzed in 14 tissues or development stages of *P. trichocarpa*, including: FM, F, M, ML, PC, G43h, YFB, ApB, AxB, REF, RTC, YMB, Xylem1, and Phloem3. Detailed information about the RNA-seq data for the 55 *PtGLK* genes are listed in Table S3. Hierarchical clustering of the heatmap showed that *PtGLK* genes had divergence expressed in a variety of poplar tissues and development stages (Fig. 9). According to the expression profiles in 14 tissues, the poplar GLK family genes were divided into seven clusters (C1-C7). The four genes (*PtGLK21*, *PtGLK43*, *PtGLK45*, *PtGLK54*) clustered in C2 showed high expression levels in Xylem1 and Phloem3 tissues. A total of 20 genes grouped in C4/C5 were highly expressed in FM, F, and M. Additionally, many genes in C3 (except *PtGLK21*, *PtGLK36*, and *PtGLK41*) displayed high expression levels in Phloem3. In contrast, the majority of the 20 genes (C4, C5) presented lower expression levels in Phloem3. Taken together, the results showed that *PtGLKs* presented diverse expression profiles in different tissues and senescence stages, providing preliminary insight into further functional exploration.

### Analysis of cis-regulatory elements in the promoter regions of PtGLK genes

**Fig. 10:**
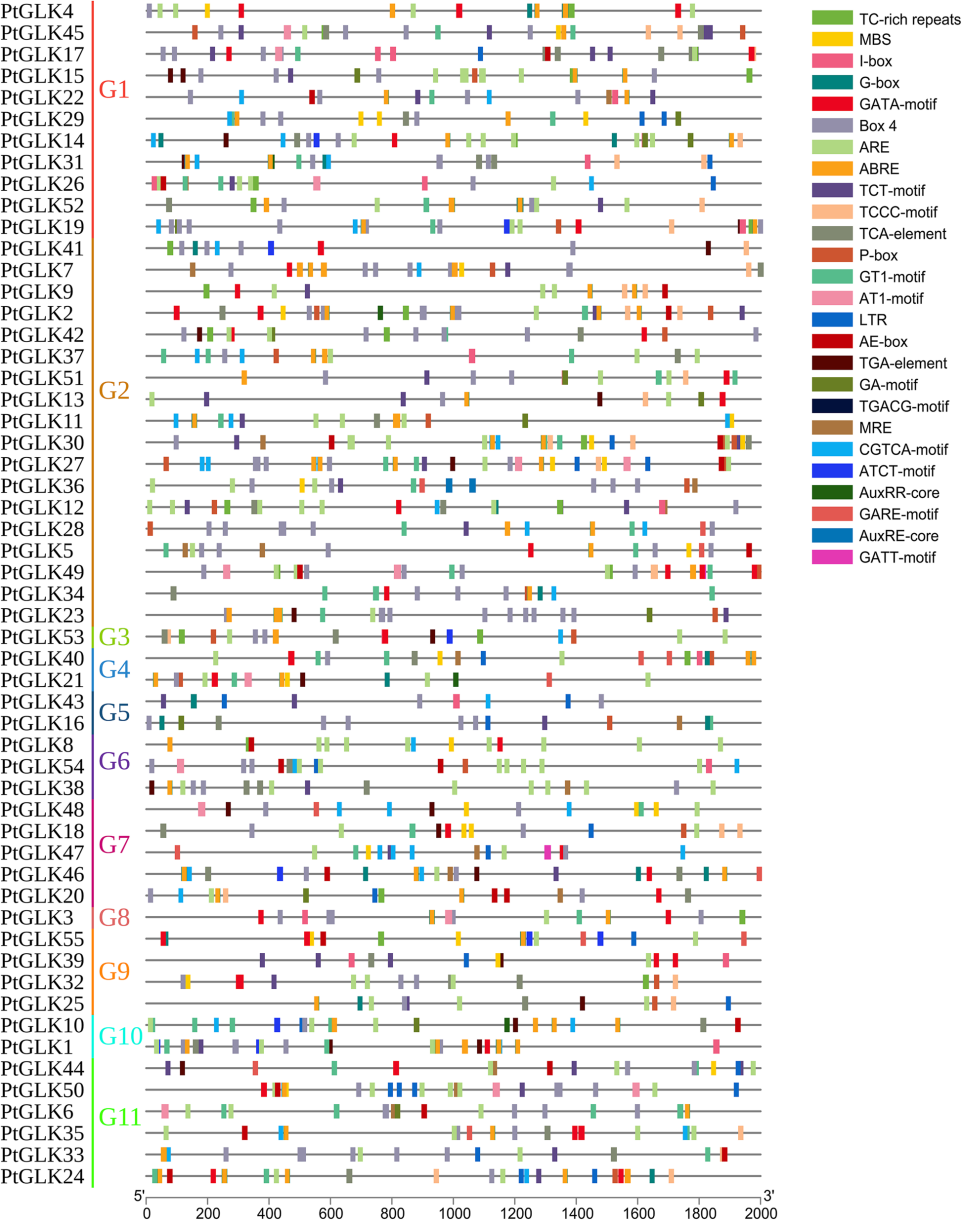
Analysis of *cis*-elements of *PtGLKs* with the Plantcare database

Analysis of the promoters of *PtGLKs* in *P. trichocarpa* revealed that various potential CREs corresponding to defense and stress, light responsiveness, cold responsiveness, osmotic responsiveness, MeJA responsiveness, GA responsiveness, IAA responsiveness, and SA responsiveness were identified[47]. Detailed elements are listed in Fig. 10 and Table S5. The numbers of CREs were also significantly different in the promoters of different poplar *GLK* gene family members. The promoters of *PtGLK30* contained the highest variety of CREs (MBS, G-box, Box4, ARE, ABRE, TCT-motif, TCCC-motif, TCA-element, P-box, GT1-motif, LTR, AE-box, TGACG-motif, MRE, and CGTCA-motif), while *PtGLK43* contained only seven kinds of CREs. Moreover, all *PtGLKs* contained one or more abiotic stress elements, this result revealed that the expression of most *PtGLK* genes was associated with abiotic stress. Additionally, a total of 36 *PtGLKs* (65.5%) had two or more phytohormone induction elements, and *PtGLK24*, *PtGLK46* and *PtGLK57* included all five phytohormone induction elements (IAA-, ABA, GA-, MeJA- and SA-) (Fig. 10). The analysis of CREs displayed that the type, quantity, and distribution of CREs in different *PtGLK* genes were dissimilar, suggesting that each *PtGLK* gene was controlled by differing groups of TFs and that the expression of *PtGLKs* could respond to different abiotic stresses and phytohormone treatments.

### PtGLK gene expression profiles in response to abiotic stress and phytohormone treatments

**Fig. 11:**
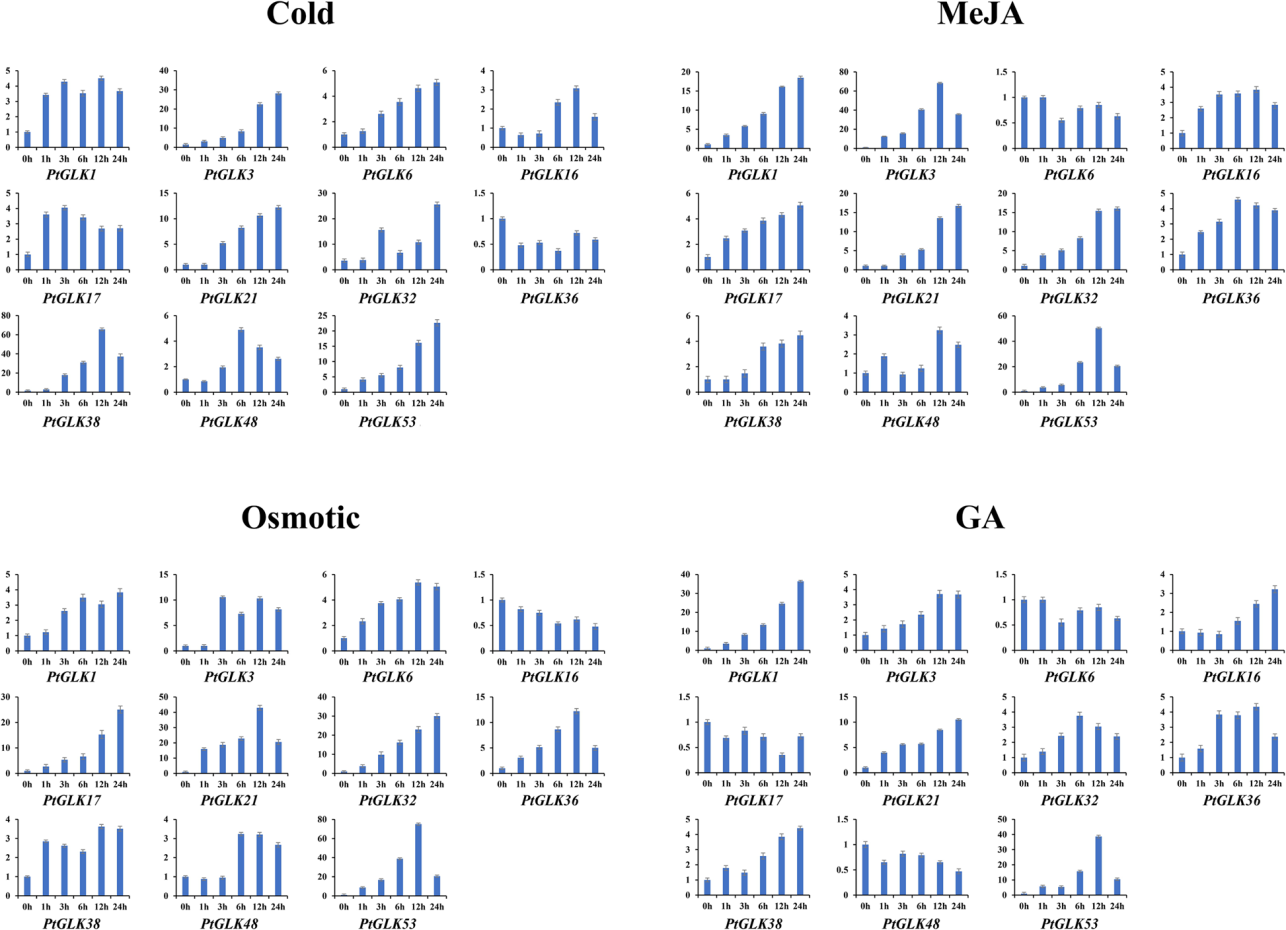
Expression patterns of 11 representative *PtGLK* genes in response to abiotic stresses and phytohormone treatments. The abiotic stresses and phytohormones used here were cold stress, osmotic stress, and MeJA and GA treatments. The relative expression levels were normalized to the reference gene *Pt18S*

Several *GLK* genes have been studied to be related to the regulation of abiotic stresses and phytohormone response in maize [25], tobacco [26], tomato [27], and moso bamboo [28]. To explore whether *PtGLK* genes also had the same function, the dynamic expression of 11 *PtGLK* genes (*PtGLK1*, *PtGLK3*, *PtGLK6*, *PtGLK16*, *PtGLK17*, *PtGLK21*, *PtGLK32*, *PtGLK36*, *PtGLK38*, *PtGLK48,* and *PtGLK53*) as representatives of each subfamily were randomly selected (Table S6). As shown in Fig. 11, there were five, six, five, and three genes, and the change of their expression levels were greater than or equal to fivefold in comparison with 0 h, showing themselves as significantly changed genes in response to cold stress, osmotic stress, MeJA, and GA treatments, respectively. Among them, *PtGLK3*, *PtGLK21, PtGLK32,* and *PtGLK53* were up-regulated both by cold and osmotic stresses, and *PtGLK1*, *PtGLK21,* and *PtGLK53* were up-regulated under both MeJA and GA treatments. In addition, *PtGLK38* (> 60-fold that of 0 h), *PtGLK53* (> 70-fold that of 0 h), *PtGLK3* (> 60-fold that of 0 h), and *PtGLK53* (> 30-fold that of 0 h) were the most highly expressed after 12 h of cold stress, osmotic stress, MeJA, and GA treatments, respectively. We also found that only the expression of *PtGLK53* was strong in response to all the four different treatments.

## Discussion

### PtGLKs in P. trichocarpa

The *GLK* genes have only been discovered in photosynthetic eukaryotes, including green algae and higher plants, and they participate in the development of chloroplasts [16, 48]. In earlier research, particular characteristics and functions of *GLK* genes were identified in *Arabidopsis* [17], maize [25], tobacco [27], tomato [26] and moso bamboo [28]. Nevertheless, the poplar GLK transcription factor has not yet been described up until now. In the current study, 55 putative *PtGLK* genes were identified in the poplar genome. The numbers of poplar GLK subfamily members were 13, 1, and 10 more than *Arabidopsis* (42), tomato (54), and sorghum (45), respectively. The greater number of *PtGLK* genes contain far more genes than those in these three species, which showed that the poplar genome size is substantially larger and is consistent with the genome duplication event [49, 50].

According to the phylogenetic analysis, the predicted poplar GLK subfamily members were classified into 11 groups (G1-G11), and all 11 groups contained different number of genes from *Z. mays* and *Arabidopsis*, suggesting that the *PtGLK* genes had diversified before these four species evolved. What is more, the absence of orthologous genes in maize G12 and G13, suggesting a divergence among *Z. mays* and *P. trichocarpa*. Moreover, *PtGLKs* belonging to the same subfamilies exhibited highly similar characteristics on the basis of their domain and gene structures, which indicated that the *PtGLKs* groupings were relatively reliable.

### Expansion of the PtGLKs suggests functional diversification

Analysis of the chromosome location showed that *PtGLKs* were extensive and in-homogeneously distributed in 19 poplar chromosomes, which could be owing to insertion, deletion, duplication, and reversion [51, 52]. Among the 55 *PtGLKs*, 22 segmental duplication events occurred, but not tandem duplication events. Segmental duplication events were the main pathway for expansion of the poplar GLK gene family. Otherwise, it has been proven that segmental duplication is more common than tandem duplication and plays a crucial role in the long-term evolution in much of the research [53–56]. The synthesis analysis of *P. trichocarpa* and *Z. mays* genome sequences made clear that there was a notable collinearity between *P. trichocarpa* and monocots maize, which coincided well with the evolutionary relationship between dicotyledons and monocotyledons.

To better explore the profiles of macroevolution and evaluate the evolutionary times in *P. trichocarpa*, the Ks and Ka for paralogous (Pt–Pt) and orthologous (Pt-At) gene pairs were evaluated. The Ks values indicated that a large-scale duplication event occurred ~ 17 MYA in *P. trichocarpa* and that the divergence times for Pt-At was approximately 118 MYA. Aggerbeck et al. showed that a whole-genome duplication event in *P. trichocarpa* occurred 12–18 MYA and the divergence time between *P. trichocarpa* and *Arabidopsis* was 102–113 MYA [57, 58]. The results of these comparisons suggest that the poplar GLK gene family went through an earlier large-scale duplication event and diversified before the separation of *Arabidopsis*. In addition, the Ka/Ks ratio can be used to define the effect of selective pressure selection on coding sequences [54]. Here, the Ka/Ks ratios for the Pt–Pt and Pt-At gene pairs were both < 1, suggesting that the *PtGLK* genes probably have went through strong purifying selection during evolution [32, 59].

### PtGLKs play an important role in poplar development

To predict possible functions of *PtGLK* genes in the growth and development of *P. trichocarpa*, we examined the expression patterns of 55 *PtGLK* genes in view of a previous reported transcriptome data. Most *PtGLK* genes showed high expression levels in xylem, which implied that they may have a function in the development of xylem. Generally speaking, compared with genes located in different subfamilies, genes in the same subfamily often have the same domains and similar functions. Previous studies show that two Arabidopsis genes (AT5G44190.1 and AT2G20570.2) were identified as functioning in leaf senescence [60]. In the current results, a total of 10 *PtGLK* genes (*PtGLK4*, *PtGLK14, PtGLK15, PtGLK17, PtGLK19, PtGLK22, PtGLK26, PtGLK31, PtGLK45,* and *PtGLK52*) were classified with the Arabidopsis GLK genes (AT5G44190.1 and AT2G20570.2) in G1 (Fig. 2), which suggested that these genes in different species were alike functionally and structurally. Therefore, it is speculated that these 10 putative *PtGLK* genes were involved in poplar Phloem3 senescence. The RNA-seq data showed that the transcript abundance of 17 *PtGLK* genes in group three decreased, which was closely related to the increase of leaf senescence level, indicating that these 17 *PtGLK* genes may play an essential role in the process of poplar leaf senescence. In addition, previous reports showed that the expression of *ZmGLK47* was high in all maize tissues and played a significant role in the formation and evolution of chloroplasts [25]. As the ortholog pair of *ZmGLK47* in *P. trichocarpa*, the PtGLK4, PtGLK14, PtGLK15, PtGLK17, PtGLK19, PtGLK22, PtGLK26, PtGLK29, PtGLK31, PtGLK45, and PtGLK52 shared the same protein structure and conserved domains and also exhibited the same expression patterns.

### Potential functions of PtGLKs in abiotic stress and phytohormone signaling responses

Plant genomes have a diversity of stress-related genes, allowing plants to respond to diverse living environments [61]. The GLK family has been reported to play a significant role in abiotic stress and phytohormone treatment response, such as cold stress, osmotic stress, salinity stress, ABA, MeJA, GA, and SA [25, 26]. Additionally, the *cis*-elements of the promoter, to a large extent, decide the stress-responsive gene expression profiles which contribute to plants adaption to disadvantages, and are associated with a variety of stimuli-responsive genes [62, 63]. Therefore, we investigated the expression of 11 selected *PtGLK* genes under the two stress treatments and two phytohormone treatments. Preliminary research showed that orthologous genes of different species were conservative in gene functions, while paralogous genes presented different functions, because of gene duplication [64]. For instance, we found that the expression of *ZmGLK1* and *PtGLK32* (the ortholog of *ZmGLK1* in *P. trichocarpa*) displayed similar patterns in response to cold and osmotic stress [25]. However, the expression of *PtGLK17* and its ortholog in maize, *ZmGLK50*, exhibited opposite patterns, which suggested that *PtGLKs* could have lost or obtained new functions during evolution (Fig. 10). These results revealed that paralogous pairs probably contribute similarly in the course of plant growth and development. In the present study, *PtGLK1, PtGLK21,* and *PtGLK53* were significantly induced in response to MeJA and GA treatments, implying that they may play important roles in the jasmonic acid and gibberellic acid signaling pathways. The expression of *PtGLK1* was induced under MeJA and GA treatments and changed only slightly under cold and osmotic treatments. In addition, there were three *PtGLK* genes (*PtGLK*6, *PtGLK16*, and *PtGLK48*) showed slight (< fivefold that at 0 h) changes in response to cold stress, osmotic stress, MeJA, and GA treatments.

## Conclusions

In this study, 55 members of the poplar GLK family were identified, which could be classified into 11 subfamilies on the basis of gene structures and conserved domains. Furthermore, the systematic analysis of chromosomal locations, synteny analysis, and evolutionary pattern offered valuable insight into the biological functions of the poplar GLK family members. The expression profiles of poplar GLK family genes indicated that *PtGLKs* were involved in various tissues and stages of poplar growth and development. The expression levels of *PtGLKs* under different abiotic and phytohormone treatments provides a basis for understanding the role of *PtGLKs* in the stress and phytohormone response. On the whole, these results will provide valuable resources to further explore the potential functional characteristics of *PtGLKs* in *P. trichocarpa*.

## Supplementary Information

**Additional file 1.**

## Data Availability

All data generated or analyzed during this study are included in this published article and its supplementary information files. The protein sequencing data of *Populus trichocarpa* for this study have been downloaded from the Phytozome12.1 database (https://phytozome.jgi.doe.gov). The putative PtGLK genes were obtained by a extensive search and alignment of previously reported Arabidopsis AtGLK1 (AT2G20570) and AtGLK2 (AT5G44190) protein sequences in the poplar genome bank. The general feature format (GFF) sequence file of *Populus trichocarpa* used in this study is available at *Populus trichocarpa* genome database (https://genome.jgi.doe.gov/portal/Poptr1/Poptr1.home.html). The raw RNA-Seq data in different tissues (FM, F, M, ML, REF, RTC, G43h, ApB, AxB, YFB, YMB, Xylem1, Phloem3, and PC) of* Populus trichocarpa* are available in the NCBI database under the Bioproject accession number GSE21481 and GSE21485 (https://www.ncbi.nlm.nih.gov/bioproject/PRJNA477910).

## Abbreviations

CDD: Conserved domain database
DBD: Myb-DNA-binding domain
DRE: Osmotic-responsive element
GA: Gibberellic acid
GLK: Golden2-like
LTRE: Cold-responsive element
MeJA: Methyl jasmonate
MW: Molecular weight
MYA: Million years ago
PEG: Polyethylene glycol 6000
pI: Isoelectric point
Ptr: *Populus trichocarpa*
SA: Silicylic acid
FM: Female catkin prior to seed release
F: Female catkins post-fertilization
M: Male catkins
ML: Mature leaf
REF: Washed fibrous roots
RTC: Roots from plants in tissue culture
AxB: Axillary bud
YFB: Newly initiated female floral buds
YMB: Newly initiated male floral buds
Xylem1: Developing xylem
PC: Phloem, Cortex, Epidermis
Phloem3: Developing phloem/cambium
