# Supplementary material for: The G2-Like gene family in Populus trichocarpa: identification, evolution and expression profiles
Source: BMC Genom Data. 2023 Jul 5;24:37. doi: 10.1186/s12863-023-01138-1 (PMC10320924; doi:10.1186/s12863-023-01138-1)
Supplement: Supplementary file 1 — Additional file 1. [file 12863_2023_1138_MOESM1_ESM.zip › Supplmental/Table S2.docx]

Table S2. The MEME motif sequences and lengths of PtGLK proteins in *P.thrchocarpa*.

| **Motif** | **Width** | **Best possible match** |
| --- | --- | --- |
| 1 | 41 | RWTPELHERFVHAVEQLGGPEKATPKSVLELMNVKGLTJYH |
| 2 | 41 | ITEALRMQMEVQRRLHEQLEVQRHLQLRIEAQGKYLQSILE |
| 3 | 15 | VKSHLQKYRLAKYRP |
| 4 | 50 | LEEERRKIDAFKRELPLCMLLLTEAIEALKEELMQCRENNNLIPVLEEFI |
| 5 | 22 | EEEKARSGSVRQYVRSKMPRL |
| 6 | 47 | ARTLKRPRLVWTPQLHKRFVDAVAHLGIKNAVPKTIMQLMNVDGLTR |
| 7 | 15 | GBSGLVLTTDPKPRL |
| 8 | 19 | DSKDKKNWMSSVQLWNQDNN |
