# Supplementary material for: The G2-Like gene family in Populus trichocarpa: identification, evolution and expression profiles
Source: BMC Genom Data. 2023 Jul 5;24:37. doi: 10.1186/s12863-023-01138-1 (PMC10320924; doi:10.1186/s12863-023-01138-1)
Supplement: Supplementary file 1 — Additional file 1. [file 12863_2023_1138_MOESM1_ESM.zip › Supplmental/Table S4.docx]

Table S4. The primers of qRT-PCR of 11 *PtGLKs* and *Pt18S*.

| **Genes** | **Primers used in qRT-PCR(5′→3′)** | |
| --- | --- | --- |
| *PtGLK1* | Forwards | TCAGTCATGGTTCCTCAGCG |
|  | Reverse | AGCCGAACCATTTGTCCTGA |
| *PtGLK3* | Forwards | TCCGGTGCTAACAATTCGGT |
|  | Reverse | ATCTGTTTGAGCTGCCTCCC |
| *PtGLK6* | Forwards | AAATACCGGCTGGGAAAGCA |
|  | Reverse | GACAGAGCAATCGCCAACAC |
| *PtGLK16* | Forwards | AGCTGGAGAGCGTTCCTAGT |
|  | Reverse | GACGCTTTTGCACCTCCATC |
| *PtGLK17* | Forwards | GCCAAGGCTTGTTTGGACTC |
|  | Reverse | CAGCCATCTGGTGTTGGTGA |
| *PtGLK21* | Forwards | AAACCCCGGGCTTGATGAAA |
|  | Reverse | TGCATGCGAAGAGCTTCAGT |
| *PtGLK32* | Forwards | TTGCCGATGCCAAGGAGATT |
|  | Reverse | ACTGCACTCCTCGTTTGTGT |
| *PtGLK36* | Forwards | GGAAATTGGTGCGCATGGAG |
|  | Reverse | TGGCTCAGTCTCAGTTGCAG |
| *PtGLK38* | Forwards | CTGTCAATCAGCTTGGGGGT |
|  | Reverse | GTAGAGGGGAAGGGTCGTCT |
| *PtGLK48* | Forwards | TGATCGGTTCGAAGAGGCTG |
|  | Reverse | TCGCAGAGAGATGGCATTGA |
| *PtGLK53* | Forwards | ACCAGTGATGCTAAGGCCAC |
|  | Reverse | TCAAGAGGGCGTTTCAAGCA |
| *PtGLK38* | Forwards | TTGTTGAGGAGCGGGTTCAA |
|  | Reverse | TGGGACTCTAGGCCTCACAA |
| *PtGLK48* | Forwards | ACGAGCAGCAGACATCAACA |
|  | Reverse | AGCTCGGCACTAGCAAAGTT |
| *PtGLK53* | Forwards | GCGTACAACCAAAACCACCC |
|  | Reverse | TCGATGAAACTCGCTGTGCT |
| *Pt18S* | Forwards | AAAATCATTGTAGGCCATTGTCG |
|  | Reverse | ACTAAATTAAGCCAGCGGGAGTG |
