# Supplementary material for: The G2-Like gene family in Populus trichocarpa: identification, evolution and expression profiles
Source: BMC Genom Data. 2023 Jul 5;24:37. doi: 10.1186/s12863-023-01138-1 (PMC10320924; doi:10.1186/s12863-023-01138-1)
Supplement: Supplementary file 1 — Additional file 1. [file 12863_2023_1138_MOESM1_ESM.zip › Supplmental/Table S6.docx]

Table S6.

(A) Expression level of *PtGLK* genes in response to cold stress.

| **Time**  **Gene ID** | **0h** | **1h** | **3h** | **6h** | **12h** | **24h** |
| --- | --- | --- | --- | --- | --- | --- |
| ***PtGLK1*** | 1 | 3.42 | 4.29 | 3.53 | 4.51 | 3.67 |
| ***PtGLK3*** | 1.43 | 3.09 | 4.89 | 8.35 | 22.43 | 28.18 |
| ***PtGLK6*** | 1 | 1.26 | 2.61 | 3.55 | 4.62 | 5.08 |
| ***PtGLK16*** | 1 | 0.64 | 0.72 | 2.35 | 3.08 | 1.59 |
| ***PtGLK17*** | 1 | 3.61 | 4.05 | 3.42 | 2.69 | 2.71 |
| ***PtGLK21*** | 1 | 1 | 5.21 | 8.23 | 10.6 | 12.2 |
| ***PtGLK32*** | 3.6 | 3.8 | 15.6 | 6.7 | 10.8 | 25.6 |
| ***PtGLK36*** | 1 | 0.48 | 0.53 | 0.37 | 0.72 | 0.59 |
| ***PtGLK38*** | 1.65 | 2.81 | 17.84 | 30.9 | 65.6 | 37.21 |
| ***PtGLK48*** | 1 | 0.85 | 1.95 | 4.89 | 3.51 | 2.62 |
| ***PtGLK53*** | 1 | 4.15 | 5.53 | 8.09 | 16.14 | 22.65 |

1. Expression level of *PtGLK* genes in response to drought stress.

| **Time**  **Gene ID** | **0h** | **1h** | **3h** | **6h** | **12h** | **24h** |
| --- | --- | --- | --- | --- | --- | --- |
| ***PtGLK1*** | 1 | 1.23 | 2.62 | 3.49 | 3.05 | 3.83 |
| ***PtGLK3*** | 1 | 1 | 10.55 | 7.28 | 10.31 | 8.15 |
| ***PtGLK6*** | 1 | 2.33 | 3.75 | 4.05 | 5.38 | 5.05 |
| ***PtGLK16*** | 1 | 0.82 | 0.75 | 0.54 | 0.62 | 0.48 |
| ***PtGLK17*** | 1 | 2.74 | 5.36 | 6.61 | 15.27 | 25.05 |
| ***PtGLK21*** | 1 | 15.92 | 18.74 | 22.81 | 42.95 | 20.56 |
| ***PtGLK32*** | 1 | 3.83 | 9.78 | 16.18 | 22.97 | 30.02 |
| ***PtGLK36*** | 1 | 3.07 | 5.12 | 8.64 | 12.21 | 5.01 |
| ***PtGLK38*** | 1 | 2.85 | 2.62 | 2.31 | 3.62 | 3.51 |
| ***PtGLK48*** | 1 | 0.88 | 0.95 | 3.23 | 3.21 | 2.67 |
| ***PtGLK53*** | 1 | 8.71 | 16.72 | 38.81 | 75.01 | 20.56 |

1. Expression level of *PtGLK* genes in response to MeJA treatment.

| **Time**  **Gene ID** | **0h** | **1h** | **3h** | **6h** | **12h** | **24h** |
| --- | --- | --- | --- | --- | --- | --- |
| ***PtGLK1*** | 1 | 3.43 | 5.82 | 9.02 | 16.15 | 18.48 |
| ***PtGLK3*** | 1 | 12.34 | 15.65 | 40.62 | 68.34 | 35.66 |
| ***PtGLK6*** | 1 | 1 | 0.55 | 0.79 | 0.85 | 0.63 |
| ***PtGLK16*** | 1 | 2.61 | 3.52 | 3.59 | 3.83 | 2.84 |
| ***PtGLK17*** | 1 | 2.47 | 3.08 | 3.86 | 4.31 | 5.05 |
| ***PtGLK21*** | 1 | 1 | 3.75 | 5.31 | 13.49 | 16.71 |
| ***PtGLK32*** | 1 | 3.75 | 5.09 | 8.26 | 15.41 | 16.02 |
| ***PtGLK36*** | 1 | 2.46 | 3.15 | 4.59 | 4.21 | 3.89 |
| ***PtGLK38*** | 1 | 1 | 1.48 | 3.58 | 3.82 | 4.47 |
| ***PtGLK48*** | 1 | 1.88 | 0.93 | 1.25 | 3.24 | 2.48 |
| ***PtGLK53*** | 1 | 3.75 | 5.71 | 23.47 | 50.41 | 20.52 |

1. Expression level of *PtGLK* genes in response to GA treatment.

| **Time**  **Gene ID** | **0h** | **1h** | **3h** | **6h** | **12h** | **24h** |
| --- | --- | --- | --- | --- | --- | --- |
| ***PtGLK1*** | 1 | 3.47 | 8.19 | 13.36 | 24.59 | 36.38 |
| ***PtGLK3*** | 1 | 1.42 | 1.72 | 2.35 | 3.71 | 3.68 |
| ***PtGLK6*** | 1 | 1 | 0.55 | 0.79 | 0.85 | 0.63 |
| ***PtGLK16*** | 1 | 0.93 | 0.85 | 1.55 | 2.45 | 3.22 |
| ***PtGLK17*** | 1 | 0.69 | 0.83 | 0.71 | 0.35 | 0.72 |
| ***PtGLK21*** | 1 | 3.98 | 5.61 | 5.69 | 8.46 | 10.52 |
| ***PtGLK32*** | 1 | 1.39 | 2.43 | 3.76 | 3.04 | 2.39 |
| ***PtGLK36*** | 1 | 1.59 | 3.84 | 3.79 | 4.35 | 2.37 |
| ***PtGLK38*** | 1 | 1.79 | 1.48 | 2.58 | 3.85 | 4.42 |
| ***PtGLK48*** | 1 | 0.65 | 0.82 | 0.79 | 0.65 | 0.47 |
| ***PtGLK53*** | 1 | 5.75 | 5.42 | 15.71 | 38.62 | 10.52 |
