# Supplementary material for: The G2-Like gene family in Populus trichocarpa: identification, evolution and expression profiles
Source: BMC Genom Data. 2023 Jul 5;24:37. doi: 10.1186/s12863-023-01138-1 (PMC10320924; doi:10.1186/s12863-023-01138-1)
Supplement: Supplementary file 1 — Additional file 1. [file 12863_2023_1138_MOESM1_ESM.zip › Supplmental/Table S1.doc]

Table S1. Detailed information about *ZmGLK* genes in maize and *AtGLK* genes in *Arabidopsis*.

| **Gene name** | **Sequences ID** | **MW(Da)** | **PI** | **CDS Length(bp)** | **Size(aa)** |
| --- | --- | --- | --- | --- | --- |
| *ZmGLK1* | GRMZM2G009060_T01 | 39861.23 | 7.28 | 1080 | 359 |
| *ZmGLK2* | GRMZM2G006477_T01 | 49645.57 | 6.16 | 1353 | 450 |
| *ZmGLK3* | GRMZM2G113742_T01 | 29156.18 | 9.43 | 804 | 267 |
| *ZmGLK4* | GRMZM2G056400_T01 | 47557.66 | 8.82 | 1377 | 458 |
| *ZmGLK5* | GRMZM5G887276_T03 | 46203.21 | 8.73 | 1305 | 434 |
| *ZmGLK6* | GRMZM2G039074_T02 | 45382.56 | 9.8 | 1284 | 427 |
| *ZmGLK7* | GRMZM2G034563_T01 | 26111.26 | 6.92 | 747 | 248 |
| *ZmGLK8* | GRMZM2G374986_T01 | 42557.7 | 9.3 | 1176 | 391 |
| *ZmGLK9* | AC234520.1_FGT003 | 19591.43 | 9.56 | 561 | 186 |
| *ZmGLK10* | GRMZM2G124540_T01 | 35804.38 | 6.12 | 1017 | 338 |
| *ZmGLK11* | GRMZM2G379656_T01 | 16687.42 | 5.15 | 438 | 145 |
| *ZmGLK12* | GRMZM2G117193_T01 | 53169.28 | 7.22 | 1488 | 495 |
| *ZmGLK13* | GRMZM2G125704_T02 | 30274.45 | 9.19 | 816 | 271 |
| *ZmGLK14* | GRMZM2G035370_T01 | 51625.62 | 7.8 | 1404 | 467 |
| *ZmGLK15* | GRMZM2G069525_T01 | 33135.25 | 9.08 | 939 | 312 |
| *ZmGLK16* | GRMZM2G173882_T01 | 36735.53 | 7.02 | 1038 | 345 |
| *ZmGLK17* | GRMZM5G846506_T01 | 41247.17 | 9.47 | 1146 | 381 |
| *ZmGLK18* | GRMZM2G370425_T01 | 43773.75 | 6.65 | 1230 | 409 |
| *ZmGLK19* | GRMZM2G348238_T01 | 52172 | 5.8 | 1500 | 499 |
| *ZmGLK20* | GRMZM2G074908_T01 | 32180.44 | 5.68 | 915 | 304 |
| *ZmGLK21* | GRMZM2G087804_T03 | 49254.78 | 6.72 | 1386 | 461 |
| *ZmGLK22* | GRMZM2G396825_T01 | 32555.42 | 8.54 | 894 | 297 |
| *ZmGLK23* | GRMZM2G175827_T02 | 49074.33 | 9.16 | 1407 | 468 |
| *ZmGLK24* | GRMZM2G070865_T01 | 39524.16 | 6.71 | 1119 | 372 |
| *ZmGLK25* | GRMZM2G315506_T01 | 37640.56 | 8.66 | 1074 | 357 |
| *ZmGLK26* | GRMZM2G168002_T04 | 28660.53 | 6.59 | 813 | 270 |
| *ZmGLK27* | AC233960.1_FGT003 | 30635.8 | 8.4 | 840 | 279 |
| *ZmGLK28* | GRMZM2G052544_T01 | 40783.49 | 7.22 | 1131 | 376 |
| *ZmGLK29* | GRMZM2G083472_T01 | 33179.87 | 5.69 | 921 | 306 |
| *ZmGLK30* | GRMZM2G010920_T01 | 48305.91 | 6.61 | 1317 | 438 |
| *ZmGLK31* | GRMZM2G477238_T01 | 58738.69 | 6.21 | 1623 | 540 |
| *ZmGLK32* | GRMZM2G060834_T01 | 40092.95 | 7.41 | 1143 | 380 |
| *ZmGLK33* | GRMZM2G106185_T01 | 40665.18 | 9.85 | 1137 | 378 |
| *ZmGLK34* | GRMZM2G060485_T01 | 58845.41 | 6.36 | 1638 | 545 |
| *ZmGLK35* | GRMZM2G379167_T01 | 44260.81 | 6.57 | 1209 | 402 |
| *ZmGLK36* | GRMZM2G171468_T01 | 38386.96 | 5.33 | 1089 | 362 |
| *ZmGLK37* | GRMZM2G701218_T01 | 46434.86 | 6.53 | 1245 | 414 |
| *ZmGLK38* | AC219020.4_FGT002 | 59226.91 | 6.17 | 1665 | 554 |
| *ZmGLK39* | GRMZM2G117854_T03 | 28416.91 | 6.08 | 774 | 257 |
| *ZmGLK40* | GRMZM2G100709_T01 | 28085.27 | 7.71 | 771 | 256 |
| *ZmGLK41* | GRMZM2G398055_T01 | 31420.01 | 7.09 | 939 | 312 |
| *ZmGLK42* | AC155434.2_FGT005 | 39792.34 | 7.78 | 1104 | 367 |
| *ZmGLK43* | GRMZM2G064197_T01 | 27732.51 | 8.48 | 771 | 256 |
| *ZmGLK44* | GRMZM2G162409_T03 | 48417.02 | 5.14 | 1326 | 441 |
| *ZmGLK45* | GRMZM2G173943_T02 | 34026.39 | 5.95 | 939 | 312 |
| *ZmGLK46* | GRMZM2G082264_T01 | 48352.37 | 6.98 | 1434 | 477 |
| *ZmGLK47* | GRMZM2G100176_T01 | 39865.83 | 7.06 | 1110 | 369 |
| *ZmGLK48* | AC234155.1_FGT002 | 44110.34 | 6.23 | 1182 | 393 |
| *ZmGLK49* | GRMZM2G067702_T01 | 23717.77 | 6.55 | 660 | 219 |
| *ZmGLK50* | GRMZM2G471600_T01 | 30828.47 | 6.83 | 900 | 299 |
| *ZmGLK51* | GRMZM2G081671_T01 | 37367.17 | 7.7 | 1032 | 343 |
| *ZmGLK52* | GRMZM2G333083_T01 | 56361.35 | 6.48 | 1566 | 521 |
| *ZmGLK53* | GRMZM2G454449_T01 | 28049.28 | 8.59 | 762 | 253 |
| *ZmGLK54* | GRMZM2G026833_T01 | 50523.81 | 5.2 | 1431 | 476 |
| *ZmGLK55* | GRMZM2G124495_T01 | 49168.55 | 8.15 | 1377 | 458 |
| *ZmGLK56* | GRMZM2G016370_T01 | 48702.17 | 6.08 | 1362 | 453 |
| *ZmGLK57* | GRMZM2G159119_T01 | 49338.97 | 6.58 | 1380 | 459 |
| *ZmGLK58* | GRMZM2G090230_T01 | 35491.89 | 8.74 | 987 | 328 |
| *ZmGLK59* | GRMZM2G123308_T02 | 37355.24 | 7.35 | 1071 | 356 |
| *AtGLK1* | AT1G13300 | 39057.22 | 6.03 | 1035 | 344 |
| *AtGLK2* | AT1G14600 | 29094.31 | 6.95 | 768 | 255 |
| *AtGLK3* | AT1G25550 | 38849.49 | 7.17 | 1035 | 344 |
| *AtGLK4* | AT1G32240 | 43840.56 | 7.45 | 1167 | 388 |
| *AtGLK5* | AT1G49560 | 37381.48 | 7.64 | 1002 | 333 |
| *AtGLK6* | AT1G68670 | 39424.22 | 6.58 | 1065 | 354 |
| *AtGLK7* | AT1G69580 | 38038.27 | 6.29 | 1014 | 337 |
| *AtGLK8* | AT1G79430 | 39597.51 | 8.73 | 1077 | 358 |
| *AtGLK9* | AT2G01060 | 31074.67 | 5.64 | 861 | 286 |
| *AtGLK10* | [AT2G02060](https://www.arabidopsis.org/servlets/TairObject?type=locus&name=AT2G02060) | 28612.68 | 6.62 | 771 | 256 |
| *AtGLK11* | [AT2G03500](https://www.arabidopsis.org/servlets/TairObject?type=locus&name=AT2G03500) | 47961.75 | 6.62 | 1299 | 432 |
| *AtGLK12* | [AT2G06020](https://www.arabidopsis.org/servlets/TairObject?type=locus&name=AT2G06020) | 33818.63 | 5.66 | 906 | 301 |
| *AtGLK13* | [AT2G20400](https://www.arabidopsis.org/servlets/TairObject?type=locus&name=AT2G20400) | 44112.23 | 4.74 | 1194 | 397 |
| *AtGLK14* | [AT2G20570](https://www.arabidopsis.org/servlets/TairObject?type=locus&name=AT2G20570) | 47944.91 | 6.13 | 1263 | 436 |
| *AtGLK15* | [AT2G38300](https://www.arabidopsis.org/servlets/TairObject?type=locus&name=AT2G38300) | 38925.22 | 8.85 | 1023 | 340 |
| *AtGLK16* | [AT2G40260](https://www.arabidopsis.org/servlets/TairObject?type=locus&name=AT2G40260) | 46231.71 | 6.29 | 1233 | 410 |
| *AtGLK17* | [AT2G40970](https://www.arabidopsis.org/servlets/TairObject?type=locus&name=AT2G40970) | 27554.43 | 9.12 | 747 | 248 |
| *AtGLK18* | [AT2G42660](https://www.arabidopsis.org/servlets/TairObject?type=locus&name=AT2G42660) | 28913.82 | 9.32 | 768 | 255 |
| *AtGLK19* | [AT3G04030](https://www.arabidopsis.org/servlets/TairObject?type=locus&name=AT3G04030) | 44612.13 | 6.69 | 1167 | 394 |
| *AtGLK20* | [AT3G04450](https://www.arabidopsis.org/servlets/TairObject?type=locus&name=AT3G04450) | 49400.23 | 5.71 | 1329 | 442 |
| *AtGLK21* | [AT3G10760](https://www.arabidopsis.org/servlets/TairObject?type=locus&name=AT3G10760) | 36114.03 | 6.48 | 1008 | 335 |
| *AtGLK22* | [AT3G12730](https://www.arabidopsis.org/servlets/TairObject?type=locus&name=AT3G12730) | 26928.67 | 8.36 | 708 | 235 |
| *AtGLK23* | [AT3G13040](https://www.arabidopsis.org/servlets/TairObject?type=locus&name=AT3G13040) | 50112.08 | 6.22 | 1350 | 449 |
| *AtGLK24* | [AT3G19070](https://www.arabidopsis.org/servlets/TairObject?type=locus&name=AT3G19070) | 37712.08 | 5.05 | 1041 | 346 |
| *AtGLK25* | [AT3G24120](https://www.arabidopsis.org/servlets/TairObject?type=locus&name=AT3G24120) | 32553.67 | 6.67 | 897 | 298 |
| *AtGLK26* | [AT3G25790](https://www.arabidopsis.org/servlets/TairObject?type=locus&name=AT3G25790) | 40369.85 | 7.60 | 1074 | 357 |
| *AtGLK27* | [AT3G46640](https://www.arabidopsis.org/servlets/TairObject?type=locus&name=AT3G46640) | 35011.56 | 5.56 | 767 | 324 |
| *AtGLK28* | [AT4G04580](https://www.arabidopsis.org/servlets/TairObject?type=locus&name=AT4G04580) | 19215.84 | 8.80 | 501 | 166 |
| *AtGLK29* | [AT4G13640](https://www.arabidopsis.org/servlets/TairObject?type=locus&name=AT4G13640) | 32115.31 | 6.61 | 888 | 295 |
| *AtGLK30* | [AT4G17695](https://www.arabidopsis.org/servlets/TairObject?type=locus&name=AT4G17695) | 36922.89 | 8.72 | 969 | 322 |
| *AtGLK31* | [AT4G28610](https://www.arabidopsis.org/servlets/TairObject?type=locus&name=AT4G28610) | 45546.32 | 5.78 | 1230 | 409 |
| *AtGLK32* | [AT4G37180](https://www.arabidopsis.org/servlets/TairObject?type=locus&name=AT4G37180) | 41678.84 | 6.09 | 1092 | 363 |
| *AtGLK33* | [AT5G05090](https://www.arabidopsis.org/servlets/TairObject?type=locus&name=AT5G05090) | 29441.14 | 7.21 | 801 | 266 |
| *AtGLK34* | [AT5G06800](https://www.arabidopsis.org/servlets/TairObject?type=locus&name=AT5G06800) | 42702.94 | 6.97 | 1128 | 375 |
| *AtGLK35* | [AT5G16560](https://www.arabidopsis.org/servlets/TairObject?type=locus&name=AT5G16560) | 45845.47 | 8.64 | 1212 | 403 |
| *AtGLK36* | [AT5G18240](https://www.arabidopsis.org/servlets/TairObject?type=locus&name=AT5G18240) | 45573.86 | 6.29 | 1209 | 402 |
| *AtGLK37* | [AT5G29000](https://www.arabidopsis.org/servlets/TairObject?type=locus&name=AT5G29000) | 46221.30 | 5.43 | 1242 | 413 |
| *AtGLK38* | [AT5G42630](https://www.arabidopsis.org/servlets/TairObject?type=locus&name=AT5G42630) | 30961.32 | 7.31 | 672 | 276 |
| *AtGLK39* | [AT5G44190](https://www.arabidopsis.org/servlets/TairObject?type=locus&name=AT5G44190) | 42167.33 | 6.13 | 1161 | 386 |
| *AtGLK40* | [AT5G45580](https://www.arabidopsis.org/servlets/TairObject?type=locus&name=AT5G45580) | 29472.17 | 8.84 | 795 | 264 |
| *AtGLK41* | [AT5G59570](https://www.arabidopsis.org/servlets/TairObject?type=locus&name=AT5G59570) | 32366.94 | 4.85 | 174 | 298 |
| *AtGLK42* | [AT5G62110](https://www.arabidopsis.org/servlets/TairObject?type=locus&name=AT5G62110) | 74749.24 | 4.14 | 2022 | 673 |
